# Abiotic stress growth conditions induce different responses in kernel iron concentration across genotypically distinct maize inbred varieties

**DOI:** 10.3389/fpls.2013.00488

**Published:** 2013-12-04

**Authors:** Catherine B. Kandianis, Abigail S. Michenfelder, Susan J. Simmons, Michael A. Grusak, Ann E. Stapleton

**Affiliations:** ^1^Department of Pediatrics, USDA-ARS Children's Nutrition Research Center, Baylor College of MedicineHouston, TX, USA; ^2^Department of Biology and Marine Biology, Department of Mathematics and Statistics, University of North Carolina WilmingtonWilmington, NC, USA

**Keywords:** effect partitioning, Fe, NAM parents, nitrogen fertilizer, drought, iron nutrition, *Zea mays* L.

## Abstract

The improvement of grain nutrient profiles for essential minerals and vitamins through breeding strategies is a target important for agricultural regions where nutrient poor crops like maize contribute a large proportion of the daily caloric intake. Kernel iron concentration in maize exhibits a broad range. However, the magnitude of genotype by environment (GxE) effects on this trait reduces the efficacy and predictability of selection programs, particularly when challenged with abiotic stress such as water and nitrogen limitations. Selection has also been limited by an inverse correlation between kernel iron concentration and the yield component of kernel size in target environments. Using 25 maize inbred lines for which extensive genome sequence data is publicly available, we evaluated the response of kernel iron density and kernel mass to water and nitrogen limitation in a managed field stress experiment using a factorial design. To further understand GxE interactions we used partition analysis to characterize response of kernel iron and weight to abiotic stressors among all genotypes, and observed two patterns: one characterized by higher kernel iron concentrations in control over stress conditions, and another with higher kernel iron concentration under drought and combined stress conditions. Breeding efforts for this nutritional trait could exploit these complementary responses through combinations of favorable allelic variation from these already well-characterized genetic stocks.

## Introduction

Micronutrient malnutrition, caused by the limited availability of essential nutrients including iron, iodine, zinc, and vitamin A, is a global problem affecting billions of individuals (Black et al., 2003). The most prevalent form of such malnutrition arises from iron deficiency, which when left untreated can result in chronic illness including fatigue, shortness of breath, iron-deficiency induced anemia, irregular heartbeat, overall ill-health, and even death (WHO Worldwide prevalence of anaemia, 1993–2005; WHO Micronutrient deficiencies, 2013). Inadequate dietary iron often goes unnoticed and results in a “hidden hunger” wherein the human body becomes depleted of building blocks required for human growth until physiological damage becomes evident and irreversible (Micronutrient Initiative - Iron: Helping Children Reach Their Full Potential). In developed countries, nutritional needs can be met through the consumption of a well-balanced diet in addition to use of vitamin/mineral supplements and food fortifiers (Lynch, 2011). When essential nutrients are supplied primarily through meals rather than supplements, inadequate nutrient intake can occur from strict consumption of nutrient-poor foods or lack of dietary diversity (Bouis et al., 2011). Efforts to enhance the nutritional density of commonly consumed staple foods have been successful through the process of biofortification, in which the amount of a particular phytonutrient is increased in edible plant tissues through selective breeding and/or biotechnological methods (Nestel et al., 2006; Bouis et al., 2011). By increasing the nutrient density of these tissues in commonly consumed crops, populations at risk for chronic malnutrition can maintain a steady nutrient supply through their daily dietary intake. Nutritional enhancement of crops through biofortification also bypasses the logistical and cost issues involved with food fortification and supplement distribution, and has been shown to be a sustainable and community-empowering nutritional intervention (Saltzman et al., 2013).

Nutrient targets are more likely to be achieved through crop breeding by having an *a priori* understanding of trait diversity and heritability, as well as a consideration of accurate selection strategies using phenotypic or molecular tools. Average kernel Fe concentration (here reported as μ g g^−1^DW, also reported elsewhere as mg kg^−1^ or ppm) across various studies is 16–25 μ g g^−1^DW (Oikeh et al., 2003, 2004; Long et al., 2004; Menkir, 2008), but has been shown to reach concentrations of up to 68 μ g g^−1^ DW in replicated trials and 159 μ g g^−1^ DW in unreplicated trials of tropical maize (Maziya-Dixon et al., 2000; Prasanna et al., 2011). This trait continuum is similar to those observed for other micronutrients such as Zn in maize; however, the upper bounds of these ranges barely meet nutritional targets set by existing biofortification programs when issues of bioavailability and portion size are considered (Pfeiffer and McClafferty, 2007). Relative to other cereal crops, maize has less diversity in grain iron concentrations than wheat and pearl millet (Ortiz-Monasterio et al., 2007; Bouis and Welch, 2010) but greater diversity than that found in rice (Kandianis, unpublished data). Studies of inheritance conducted in various cereals have shown that iron concentration of cereal grain is controlled by multiple genes (Garcia-Oliveira et al., 2009; Simic et al., 2012) and that wild germplasm may harbor rare allelic variation influencing this trait (Chatzav et al., 2010). These results suggest that the existing trait range could be increased in varieties with high agronomic performance through the introgression of targeted kernel iron loci from specific donor genotypes. Varietal evaluation for kernel iron density has shown that highly variable trait heritability is caused by extensive genotype by environment (GxE) interactions, particularly when conventional agronomic inputs are unavailable or soil Fe is largely unavailable as in many areas where biofortified crops are most needed (Long et al., 2004). From a logistical standpoint, the dependence of kernel iron concentration on environmental growth conditions seemingly requires mineral nutrient trait-specific breeding programs to perform location-specific phenotypic evaluations in order to achieve the desired nutritional phenotype. From a biological perspective, this observation implies that distinct genetic networks operate under highly variable growth conditions to regulate the environmental plasticity of kernel iron concentration.

GxE interactions have been considered a hindrance to crop improvement for yield, quality, and nutritional traits alike. Even for phenotypes where moderate heritability is observed across environments, exposure to abiotic stress can substantially reduce heritability and trait stability to the point where breeding programs will make negligible gains from selection (Ceccarelli, 1989). To improve the probability of success where environmental variability is high, the use of molecular and phenotypic selection criteria tailored to specific environmental stressors is useful. In order to better understand the interaction of abiotic stress and genotypic variation in regulating kernel nutrient density, we examined the response of kernel iron concentration to variable water and nitrogen availability in a managed-stress field experiment across a set of genotypically diverse and well-characterized maize inbred lines (Flint-Garcia et al., 2005; Yu et al., 2008). Our experimental objectives were to: (1) identify generalized responses of kernel iron concentration across variable growth regimes in a set of highly diverse and well-characterized maize germplasm and; (2) determine if other mineral concentrations mediated the behavior of kernel Fe status in these lines across treatment regimes.

## Methods

### Seed stocks

Corn (*Zea mays*) seed stocks for the Nested Association Mapping (NAM) founder inbred lines (*n* = 25) were provided by Dr. James B. Holland, USDA-ARS and NCSU (McMullen et al., 2009). Seed stocks were increased using standard nursery agronomic practice in the field nursery at Central Research, Clayton, NC. Commercial hybrid seed was supplied by Central Research staff. Nursery and experimental fields were hand-pollinated.

### Field experimental design

Managed stress treatments were administered across four sectors of a leveled field composed of uniform soil (Norfolk loamy sand) in a 2 × 2 factorial design with factors being water irrigation and nitrogen application, as described for the same experiment in Makumburage and Stapleton (2011). Treatment sectors included: (1) control: irrigation throughout the growth season and with nitrogen fertilizer; (2) drought: absence of supplemental irrigation from anthesis until maturity; (3) low-nitrogen: absence of nitrogen fertilizer application and; (4) combined stress: absence of supplemental irrigation as in (2) and absence of nitrogen fertilizer application as in (3). At this site, water supply is administered through an overhead sprinkler irrigation system from stage V9 through maturity. Drought stress is prevented or imposed through water application by field staff using a combination of criteria including visible plant stress (leaf rolling), soil moisture conditions, and pond reservoir height. Climate conditions in 2008 at this site are available from the on-station weather records at http://www.nc-climate.ncsu.edu/cronos/index.php?station=CLAY. Nitrogen fertilizer was applied to the appropriate field sectors at a rate of 54 kg ha^−1^ prior to planting. Based on preseason soil test recommendations, the no nitrogen field sectors were supplemented before planting with potassium (160 kg ha^−1^), and the plus nitrogen sectors were supplemented with phosphorous (54 kg ha^−1^), potassium (160 kg ha^−1^), and sulfur (43 kg ha^−1^). Plus nitrogen sectors received two additional liquid nitrogen treatments during the growth season as band application between rows, as is standard field practice for corn at this location. Routine soil tests (http://www.ncagr.gov/agronomi/sthome.htm) taken from each sector at the end of the season showed no difference in P, K or trace nutrients between nitrogen and no-nitrogen plots. Seeds of a given genotype were hand-planted in each sector at 0.25 m spacing with one meter between each genotype. Sectors were separated from adjacent plantings with commercial hybrid border plant rows. Ears from all genotypes were concurrently harvested four months after planting when all plants had senesced and dried.

### Measurement of kernel and ear traits

Four traits including kernel weight, cob length, cob diameter, and kernel width were assayed. For measurement of kernel weight, bulks of one hundred kernels were sampled from each ear of every genotype-treatment combination. Cob diameter was assessed for all harvested ears in each entry, and was measured as the diameter of the unshelled ear. To determine kernel width, 10 individual kernels from each entry were measured at the widest point with digital calipers. Kernel data are listed in Datasheet 1.

### Maize kernel elemental analyses

Each genotype-treatment combination was represented by a single sample from one ear with the exception being genotype B73, from which three ears were sampled per treatment with two samples per ear. Twenty kernels from the middle third of the ear were collected, weighed and ground to fine powder in a stainless steel coffee grinder. To check that sample processing does not contribute to artificial Fe contamination, an external NIST reference cereal standard (rice flour) was included within each digestion set. Ground samples were dried at 65°C for 36 h prior to weighing 0.25 g per sample per digestion into borosilicate glass tubes. Digestions were performed in triplicate as in Waters and Grusak (2008); Waters et al. (2009) with minor modifications. Samples were pre-digested in 3 mL nitric acid (70%, Trace Metal Grade, Fisher Scientific) overnight, and then digested for an additional 3 h at 125°C. A 1.5 mL addition of H_2_O_2_ (30%, Fisher Scientific) with incubation at 125°C for 1 h was followed by a second 1.5 mL addition of H_2_O_2_ with incubation at 125°C for another hour. Temperature was increased to 200°C and glass tubes were evaporated to dryness. Residual mineral precipitates were dissolved in 10 mL nitric acid (2%), and were collected for subsequent analysis by ICP-OES (Inductively Coupled Plasma-Optical Emission Spectrometry). Mineral concentrations in (μ g mineral) (g sample dry weight)^−1^ were calculated for Fe and Mg, and are included in Datasheet 1.

### Data analysis

In order to examine the iron concentration (as measured by ICP analysis) and kernel weights jointly, we partitioned the data set using the R program mvpart using Euclidian distances (De'ath and Team, 2012) for both kernel iron concentration and kernel weight (using 100 kwt) as Y variables, with inbred genotype and environment as partition levels. Partition trees with scaled average trait values for each split were output from R; R code for the analysis is provided in Datasheet 2 and output of the mvpart analysis is provided in Datasheet 3. Genotype, managed growth environment, cob and kernel features, and mineral concentrations were included in a multivariate analysis using the SAS v. 9.1 Enterprise Miner module to identify variables that best predicted grain iron concentration. Correlated variables were identified using clustering (SOM/Kohonen). The best predictors of iron concentration were chosen using regression and decision tree analyses, and were then combined using the SAS Enterprise Miner Ensemble node. Significant predictor variables were used as factors in an ANOVA and in a recursive partitioning analysis. ANOVA and univariate recursive partition analyses were carried out using SAS JMP v.7 with default parameters. ANOVA output and LS means estimates are reported in Datasheets 3, 4 for two factor (genotype, treatment) and three factor (genotype, treatment, Mg concentration) analyses, respectively.

## Results

**Figure 1:**
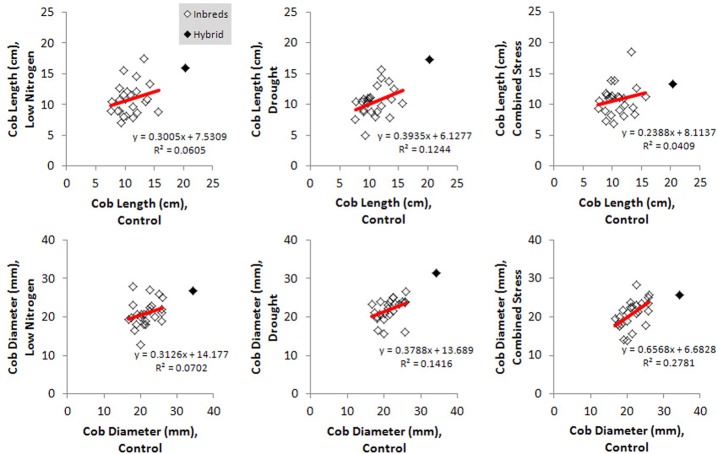
**Physiological responses to imposed environmental stress for all genotypes**. Effect of environmental stress treatments on cob length and cob diameter are shown relative to the control treatment. Responses for inbred genotypes (open marker) or the hybrid genotype (solid marker) are depicted, with regression lines drawn in red.

**Figure 2:**
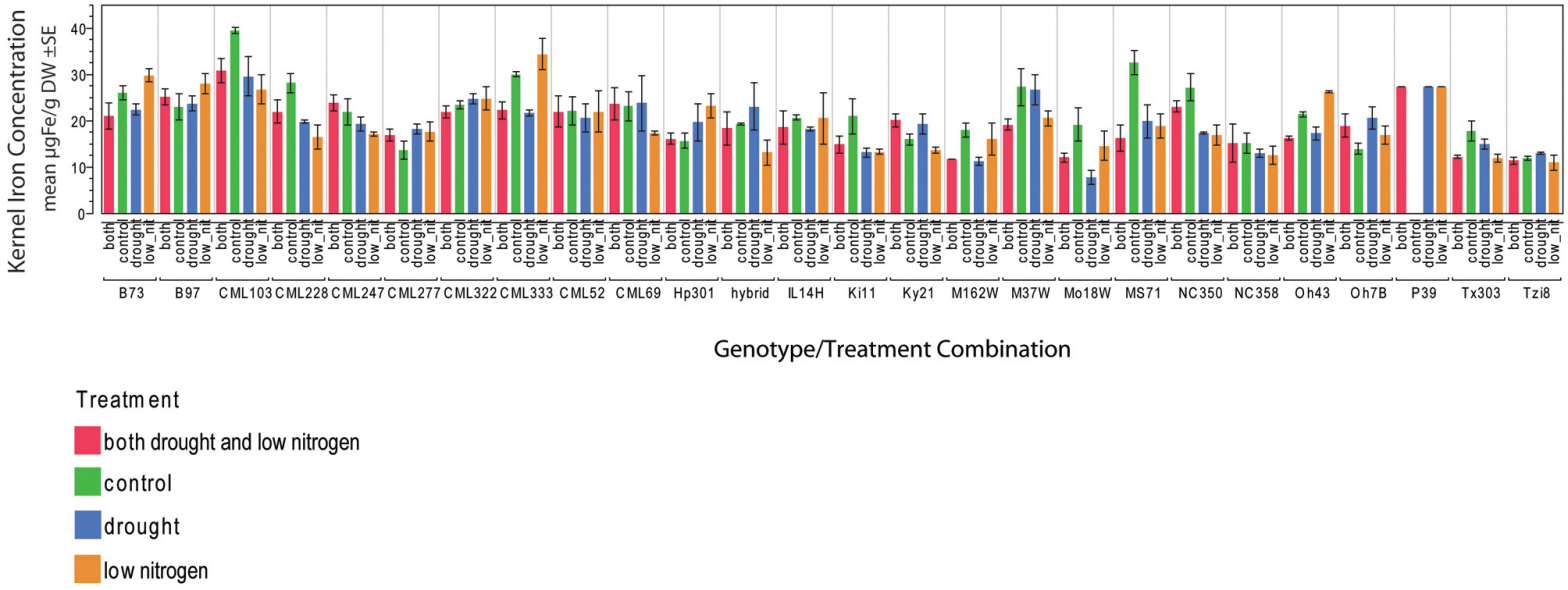
**Kernel iron concentrations by genotype-treatment combination**. Iron concentration data are represented in μ g Fe g^‒1^ DW as an average of three technical replicates.

Kernel Fe concentration for 25 inbreds and one hybrid maize line were analyzed across four managed stress environments including: control (water plus nitrogen at normal levels), low nitrogen, drought, and combined low nitrogen plus drought. Stress treatments had a negative effect on physiological growth relative to the control treatment as observed in comparisons of cob length and cob diameter (Figure 1). A linear regression of cob length or diameter on treatment effect revealed regression coefficients significantly less than 1 (cob length β 1 = 0.2388 − 0.3935; cob diameter β 1 = 0.3126 − 0.6568), indicating that both cell growth and expansion were attenuated upon imposition of drought, low nitrogen or combined stress. Kernel iron concentrations ranged from 9–37 μ g g^−1^ DW across all genotype-treatment combinations. Intra-treatment variation in kernel Fe concentration, represented by within-ear or within-row sampling of genotype B73, was non-significant and substantially less than variation among treatments (*P* = 0.068, Table 1). Genotypes with the highest grain Fe concentration across all treatments included CML333 and CML103, and genotypes with the lowest grain Fe concentration included NC358 and TZI8 (Figure 2, *P*-values, effect estimates, and pairwise comparisons given in Datasheet 4). Average kernel Fe concentration and standard errors by environmental treatment measured 22.1 ± 0.9 μ g g^−1^ DW in control, 20.2 ± 1.0 μ g g^−1^ DW in low nitrogen, 19.4 ± 0.9 μ g g^−1^ DW in drought, and 19.9 ± 0.9 μ g g^−1^ DW in combined stress (Datasheet 4). Kernel Fe concentration among the genotypes varied significantly when compared across the managed stress environments (analysis of variance model with treatment nested in genotype, *r*^2^ of 0.63, rmse of 5.14, model *P* < 0.0001, genotype [treatment] *P* < 0.0001, Datasheet 4). Twenty-three genotype-treatment combinations had environmental effects significantly different from the treatment average (Datasheet 4). Kernel Fe concentration was significantly reduced under environmental stress conditions relative to control treatment for the MS71 (*P* = 0.009) and B73 (*P* = 0.008) inbred lines (Datasheet 4). Changes in kernel Fe concentration across the tested lines were accompanied by slight differences in average kernel weight, measuring on average 27.5 ± 1.28 g (100 kernels)^−1^ in control, 26.2 ± 1.34 g (100 kernels)^−1^ in low nitrogen, 26.7 ± 1.31 g (100 kernels)^−1^ in drought, and 24.5 ± 1.34 g (100 kernels)^−1^ in combined stress. Combined stress treatment led to a 10% reduction in kernel weight over the control treatment (*P* = 0.1). Low nitrogen and drought treatments reduced average kernel weight as compared to the control treatment, but the reduction was not found to be statistically significant when all genotypes were considered.

**Table 1:** **Sources of variation for kernel iron concentration in inbred genotype B73 field samples**.

**Source of sample variation**	**DF**	**Mean squares**
Treatment	3	84.89^(^†^)^
Within row	2	11.34 (ns)
Within ear (Row)	3	26.70 (ns)
Treatment, within row interaction	6	43.94 (ns)

Sources of variation in Fe concentration were identified through comparison of treatment, within row (variation across ears), within ear (variation within an ear, nested within row), and interaction effects in genotype B73. With the exception of environmental treatment

† (p = 0.068), all sources of sample variation were not found to have an effect on kernel Fe concentration.

**Figure 3:**
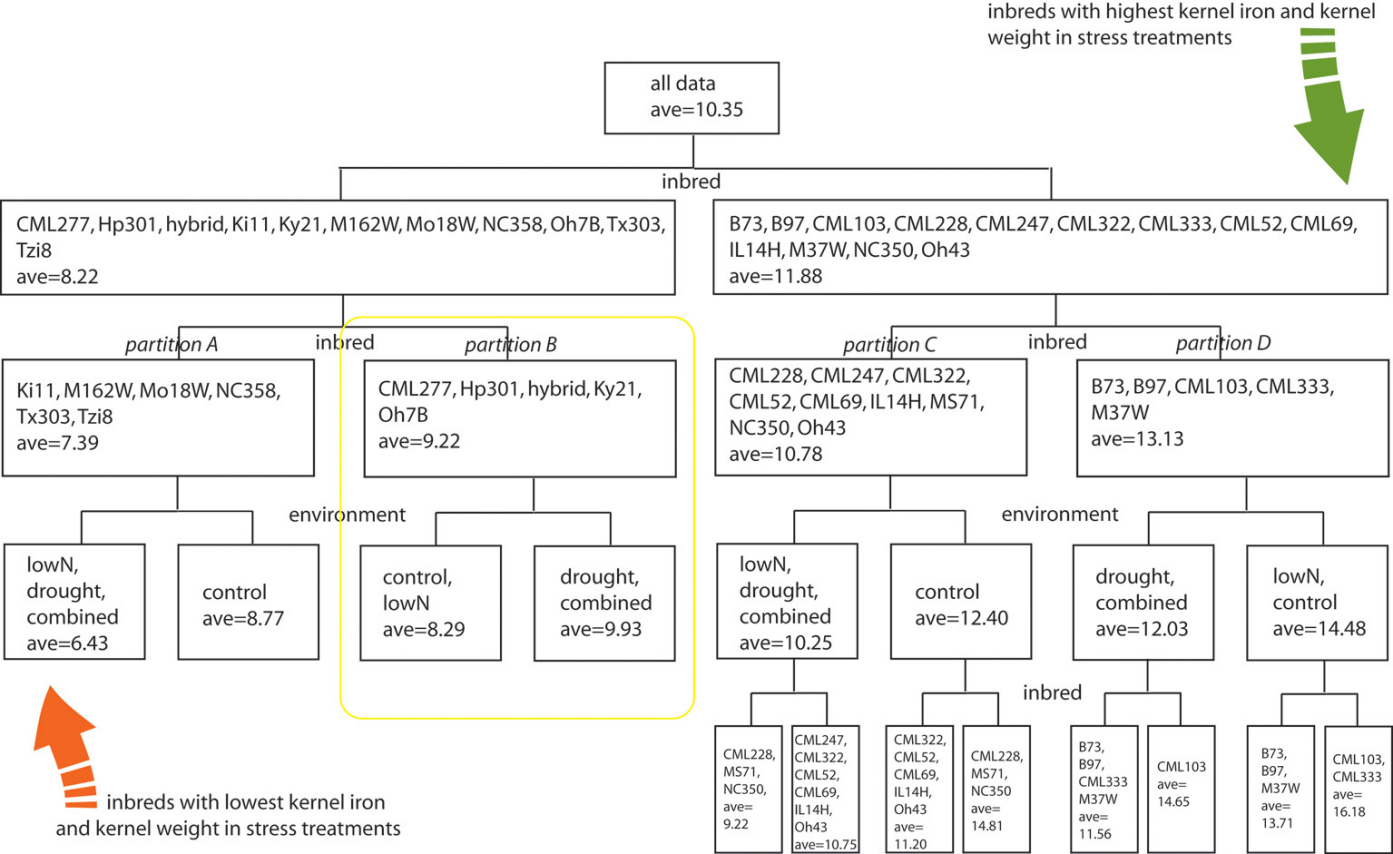
**Multivariate partition analysis of inbred and environmental treatment factors important for kernel iron concentration**. Combined response variables representing kernel Fe concentration and weight from each genotype were partitioned into subclasses defined by either inbred or environmental treatment conditions. Partitions were used to maximize differences in the combined Fe-weight response variable, leading to hierarchical splitting of genotype-treatment combinations until response variables were no longer considered significantly different among partition members. Factors used to distinguish subclasses are listed at the base of the node or data split; members of each partition are indicated within the box by levels of the splitting factor. Average values represent a combined kernel Fe concentration and weight variable, generated by partition analysis as indicated in methods section. The four inbred partitions representing genotype groupings with similar kernel Fe concentration and weight responses to environmental treatment are indicated with italic labels as partitions **A**, **B**, **C**, and **D**.

To evaluate how growth environment and genotype could affect kernel iron concentration, we performed a multivariate partition analysis across all treatment/genotype combinations. Kernel biomass has been shown to dilute or exaggerate the magnitude of kernel mineral concentration (Feil et al., 2005). Accordingly, we generated a kernel weight and iron concentration inclusive response variable, on which we performed partitioning analyses to find the genotype and treatment grouping(s) that would best explain variation in the combined response variable (*Y*-value). The range of iron concentrations and kernel weights measured was partitioned into 4 hierarchical levels using a total of 11 significant splits, with seven splits for inbred and four for treatment. Inbred genotype groupings defined the initial data split, leading to partitions **A**, **B**, **C**, and **D** (Figure 3, first and second levels). Subsequent differences in the response variable were found to partition according to environmental treatment (third level). Inbreds with the lowest Fe concentration in stress treatment environments are Ki11, MO18W, M162W, NC358, Tzi8, and Tx303 (Figure 3, Partition **A**), while the inbred with the highest Fe concentration and kernel weight in stress environments are CML103 and CML333 (Figure 3, subgroup of Partition **D**). Kernel Fe concentrations and kernel weights were higher under control conditions than stress treatments for all genotypes except those in partition **B** which include CML277, Hp301, Ky21, Oh7B, and the commercial hybrid (Figure 3, yellow box). Unlike all other partitions, lines in partition **B** maintained higher Fe concentrations and kernel weight in the drought and combined stress environments over that observed under control or low nitrogen conditions.

**Figure 4:**
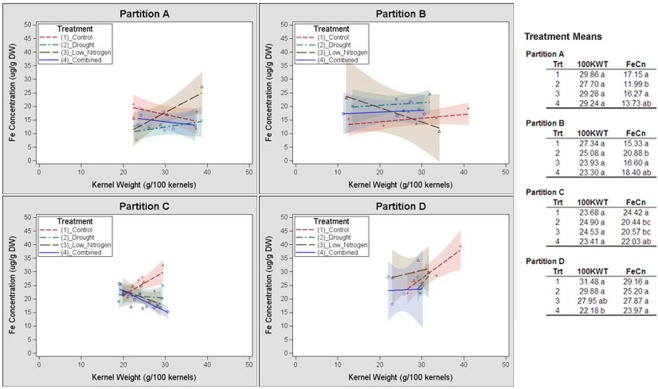
**Genotype partition-specific response patterns of kernel weight and iron concentrations to stress environment**. Kernel weight (100 KWT) and iron concentration for members of partition groups **A**, **B**, **C** and **D** in Figure 3 are plotted by environmental treatment. Relationship of kernel weight and kernel Fe concentration among all members of a partition group for a single treatment is demarcated by a linear regression. Regressions are flanked by a 90% confidence band. Average kernel iron concentrations and kernel weights from all members within a partition by environmental treatment are shown in the top right corner of each panel.

**Figure 5:**
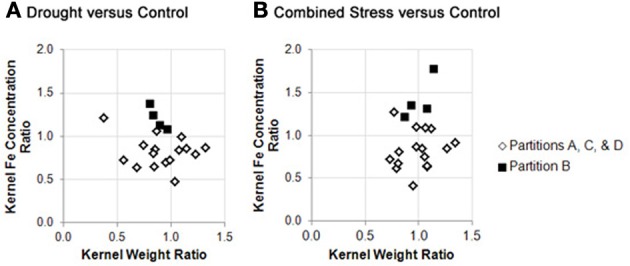
**Stability of kernel weight and iron concentration under stress conditions across genotype partition groups**. Fold change in kernel weight (X variable) or kernel iron concentration (Y variable) between environmental treatments are plotted as trait ratios (stress: control) for all genotypes in this study. Ratios are shown for: **(A)** drought vs. control, and; **(B)** combined stress vs. control. Genotypes from partitions **A**, **C**, and **D** are represented by open markers, and genotypes from partition **B** are represented by closed markers. Due to missing kernel weight data, trait ratios are not presented for all genotypes. (Genotypes with missing data points can be found in Supplementary File 1).

Germplasm partition groups, the second level of partition in Figure 3, were further characterized by the relationship of kernel weight and kernel Fe concentration as depicted in Figure 4, with partition **A** lines exhibiting high kernel weight (20–40 g/100 kernels) and low kernel Fe concentration (5–25 μ g g^−1^DW), partitions **B** and **C** demonstrating variable kernel weight (10–40 g/100 kernels) with moderate kernel Fe concentration (15–30 μ g g^−1^DW), and partition **D** showing high kernel weight (20–40 g/100 kernels) with high kernel Fe concentration (20–40 μ g g^−1^ DW). The effect of stressed growth conditions relative to the control environment led to an overall reduction in kernel Fe concentration for germplasm in partitions **A**, **C**, and **D**, but not **B**. Kernel iron concentrations for partition **B** genotypes were constant or even improved under drought or combined stress growth conditions relative to control conditions (Figure 5, seed Fe concentration ratio >1.0), whereas genotypes from other partitions incurred a reduction in kernel iron with stress exposure. Interestingly, the iron trait in partition **B** genotypes largely improved without a reduction in kernel weight (Figure 4, seed weight ratio >1.0), suggesting that both kernel iron nutrition and kernel biomass could be simultaneously maintained or improved under drought or combined abiotic stress. Increased kernel Fe concentration was coincident with increased kernel weight for some treatments, such as the low nitrogen treatment in partition **A**. However, other treatment-partition combinations demonstrated an inverse relationship between kernel weight and kernel Fe concentration, such as the low nitrogen treatment in partition **B**.

We posited that kernel iron concentration could be dependent on factors in addition to growth environment and genotype, including kernel and ear morphology traits or other measured mineral concentrations (see methods for full trait list). Variable selection with combined regression and decision trees using SAS Enterprise Mining analysis methods resulted in selection of three variables accounting for significant variation in kernel iron concentration: inbred genotype, treatment type, and Mg concentration. Genotype nested within treatment environment and pedigree interactions with Mg concentration are highly correlated with iron concentration in maize kernels when those factors are fit with ANOVA models [model *r*^2^ of 0.72, RMSE = 4.82, *P* < 0.0001 for model, *P* < 0.0001 for genotype (treatment) and *P* = 0.015 for genotype^*^Mg concentration]. Effect size and additional model details are provided in Datasheet 5.

Kernel iron and magnesium concentration across all experimental treatments were positively correlated (*r* = 0.3162, *P* < 0.0001), with the correlation still high and significant in control (*r* = 0.3423, *P* = 0.0056), low-nitrogen (*r* = 0.4267, *P* = 0.0004), and combined stress (*r* = 0.3146, *P* = 0.009) treatments (Datasheet 6). As iron concentration is affected by inbred-treatment interactions and inbred-magnesium concentrations, we partitioned iron concentration with all three factors—treatment, inbred type, and Mg concentration (Presentation 1). The partition analysis had 40 splits, with 18 for inbred, 12 for treatment, and 10 for Mg, resulting in an improvement in the amount of phenotypic variation explained (overall *r*^2^ of 0.688). Relative to treatment, magnesium concentration and inbred type defined higher order partitions of the kernel Fe concentration data (Presentation 1, top). Genotypes representing the lower and upper tails of the kernel iron concentration distribution in the Mg-inclusive partition analysis (Presentation 1) were placed similarly in the Mg-independent partition analysis (Figure 3).

## Discussion

Trait improvement resulting from response to selection is dependent upon the heritability of the trait, and the extent to which phenotypic variation is modified in the selected population over that found in the initial breeding pool (Lynch and Walsh, 1998). Most breeding programs that emphasize improvement of grain nutrient density have opted to capitalize on phenotypic extremes, using phenotypic selection to choose parent genotypes from a broad range of germplasm (Bänziger and Long, 2000; Beebe et al., 2000; Gregorio et al., 2000; Monasterio and Graham, 2000). Despite the existing range in variation for the kernel iron trait, efforts to improve this trait through additional phenotypic selection have been thwarted by low heritability. Some have found little to no GxE effect for the trait (Menkir, 2008; Lung'aho et al., 2011; Simic et al., 2012) but still others have observed that parent genotype responses from a single environment are inadequate predictors of performance across highly variable environments (Ahmadi et al., 1993; Oikeh et al., 2003, 2004; Long et al., 2004; Chakraborti et al., 2011; Prasanna et al., 2011). To characterize the nature of the GxE effects for kernel Fe concentration, we categorized a set of inbred lines according to the scale of their response in kernel Fe concentration to abiotic stress. Single and combined stress environments induced dissimilar effects on iron density and kernel weight as observed by distinct treatment groupings across multivariate partition analyses (Figure 3, Presentation 1). The effect of drought treatment on Fe concentration was, however, more frequently grouped with that of combined stress, while low-nitrogen effects were coincident with the control treatment. This may reflect the degree of independence of the genetic networks for these stress types, with drought networks having a larger or upstream effect as compared to nitrogen limitation response networks.

Among the 25 lines evaluated in this study, a response characterized by higher grain Fe concentration in adequate water and nitrogen growth conditions relative to regimes with insufficient inputs was observed in 20 genotypes grouped in partitions **A**, **C**, and **D**. This observation supports observations from a multi-environment, multi-stress field study of tropical breeding lines (Long et al., 2004) in which reduced water or nitrogen availability resulted in highly variable (and often lower) kernel nutrient quality. Two lines from this response group, CML333 and CML103, were consistently classified as high kernel Fe genotypes in each of the tested environmental treatments, despite a reduction in iron concentration under stress. Although yield penalties are incurred by partition **D** genotypes under stress conditions relative to control conditions, these lines exhibit the highest kernel Fe concentrations of all tested genotypes and therefore could also be favorable genetic donors for grain Fe yield. A second pattern of kernel Fe response was observed in partition **B** genotypes, in which higher Fe concentration was associated with drought and combined stress relative to control and low nitrogen conditions. Lines exhibiting this behavior did not experience a significant reduction in kernel size from control to drought/combined stress treatments relative to lines requiring optimal growth conditions for maximal kernel Fe concentration. The positive response of lines in partition **B** suggests that kernel Fe concentration can be increased under stressful environmental conditions without incurring a yield penalty, thus arguing for an independent/additive genetic basis for Fe and kernel yield stability under environmental stress. Although it has not been directly manipulated, the inverse correlation between grain micronutrient density and yield in various cereals has been observed by many groups (Bänziger and Long, 2000; Monasterio and Graham, 2000; Garvin et al., 2006). The term “dilution effect” has been used to describe the disproportional accumulation of kernel minerals relative to that of kernel biomass, leading to a reduction in kernel mineral density in larger grains (Feil et al., 2005). Strong correlation between kernel size and yield in some cereal species (Calderini and Ortiz-Monasterio, 2003; Carena et al., 2010) has led to the inference that improvements in grain nutrient density would come at a cost to yield gains. This yield penalty may not be common to all cereals (Gregorio et al., 2000). Feil et al. (2005) found that in a multi-year, drought stress by nitrogen application trial conducted on four tropical maize varieties, yield losses were coincident with pre-anthesis drought or reduced nitrogen application; however, variation in abiotic stress did not yield a consistent directional effect across all minerals. While N input either diluted (Zn, Ca) or augmented (Mn, Mg) the grain mineral density, drought was not found to have an effect on mineral traits in Feils' study. Although mineral traits examined did not overlap between the two studies, we found abiotic stress to have the opposite effect on average grain Fe concentration, with N input having no effect on kernel Fe concentration and drought/ combined stress leading to a reduction in the trait. The difference in these results may lie in the timing of the abiotic stress. Interestingly, Feil et al. (2005) found more variation in the mineral density trait among genotypes than among N treatment levels, suggesting that the genotype effect for mineral concentration in response to stress conditions drove most of the variation in that study.

Results presented in our study suggest that the extensive variation in kernel mineral concentrations across maize genotypes in response to abiotic stress can be captured through partitions or clusters that represent conserved patterns of yield and nutrient density (Figure 3). This method of classification more easily permits germplasm selection for grain Fe nutritional improvement on the basis of trait stability (maintaining a micronutrient concentration and yield range across varying inputs) or trait adaptability (trait performance being dictated by specific environment). From the existing survey of grain Fe concentration responses, it is evident that genotypes from partition **D** would most heavily contribute to the stability of mineral density across environments, particularly as (1) Fe concentration means in D are highest for each of the environmental stresses relative to those in A, B, and C, and; (2) kernel weight still remains high, despite environmental stress-induced losses. Adaptability for both the Fe and kernel weight traits is observed in partition **B**, where kernel Fe concentration and weight are highest in drought and combined stress treatments over that of control and low nitrogen treatments.

Previously published studies have revealed that genotype controls a significant portion of the variation in the mineral phenotype of cereal grains (Baxter et al., 2013). Varieties with higher nutrient density are likely to contain allelic variation influencing the iron phenotype. Accordingly, allelic variation underlying higher grain nutrient density could be explored through genetic mapping in populations derived from this small genetic panel, such as the recombinant inbred (RI) populations of the maize NAM panel (McMullen et al., 2009). Based on the kernel Fe responses observed here, informative crosses can be selected from the densely genotyped NAM population, permitting mapping resolution at the kilobase scale with reasonable power to detect QTL for a polygenic trait. The genetic basis for such traits could be comprised of loci directly involved with the kernel Fe phenotype (through uptake, transport, partitioning, etc.), or indirectly involved with the iron phenotype through effects on iron homeostasis and growth under stress conditions, both of which are observed in the mapping studies of Benke and Stich (2011), Baxter et al. (2013), and Simic et al. (2012). Observations from this study suggest that while a genetic framework of loci may constitutively underlie kernel Fe concentration, the phenotype is likely controlled by distinct gene networks as environmental stresses are varied.

The concentrations of most grain minerals are correlated in cereal grains (Waters and Grusak, 2008), as their accumulation is dependent upon the same physiological processes including acquisition from the rhizosphere, translocation through the xylem to developing leaves, remobilization through vegetative tissues and ultimate redirection (or *de novo* direction) toward developing kernels through phloem transport. However, the strength of correlation specifically between grain Mg and Fe concentration under variable abiotic stress found in this study was unexpected. The importance of both elements in cellular metabolism and photosynthesis is undisputed. In leaves, most Mg is involved with protein synthesis and in metabolic reactions where it is a co-factor of ATP; a substantial, but smaller amount is integral to tissue chlorophyll (Kirkby and Mengel, 1976; Karley and White, 2009). Iron plays a diversity of roles in cellular redox chemistry, as a metal cofactor, and as a component of chlorophyll synthesis. Mg and Fe kernel reserves are maintained to aid in redox reactions during germination, and to provide a supply as emerging seedlings become photosynthetically competent (Lobreaux and Briat, 1991). Although the nature of the relationship between variation in Fe and Mg is unclear, it is evident that these two grain minerals track nitrogen and water availability. Under low to no nitrogen availability, yield reduction is incurred across maize inbred lines as a result of reduced leaf area index, reduced leaf expansion and accelerated senescence (Uhart and Andrade, 1995; D'Andrea et al., 2006). A senescence induced program involving NAM-B1, a NAC transcription factor underlying the grain protein concentration QTL GPC-1 in wheat, has been shown to coordinate movement of N and Fe leaf stores to developing kernels through remobilization (Uauy et al., 2006; Waters et al., 2009), such that remobilization of Fe coincides with that of N. Remobilization of Mg in some, but not all, plant species has been observed to also accompany N (Hocking, 1994; Himelblau and Amasino, 2001) suggesting that the quantity and timing of N translocation from leaf sources to kernel sinks could be critical to both the protein quality and mineral density of grains, and that nitrogen availability cements the link for concurrent source-sink movement of various minerals.

Selection in the non-target (or favorable, control) environment could be useful for trait improvement if: (1) component sub-traits are used for selection, and; (2) phenotypic variability is maximized in the non-target environment (Hall and Richards, 2013). In the case of grain mineral nutrient density and abiotic stress, it is probable that lines high in kernel Fe concentration such as CML103 could have been selected in a non-target, control environment. Under control or stress conditions, they consistently yield grain iron concentrations at the upper-most extreme of the phenotypic distribution. However, without the comparative stress treatments, one could not easily predict the average reduction in grain Fe for such a line. In this study, a comparative environmental analysis by recursive partitioning enabled the identification of lines with higher kernel Fe in stress environments than control environments such as those in partition **B** (Figures 3, 4). It is worth noting that these lines would not have been selected as possible Fe trait donors on the basis of Fe concentration alone as they are not phenotypic extremes; rather, their selection is a result of the Fe concentration pattern that emerges from cross-environment comparisons. Use of partitioning on cross-environment phenotypes indicated that stress responses for the iron concentration trait are not necessarily additive in that combined-stress treatment did not consistently group with both or with a single stress environment across the four germplasm partitions (Figure 3). This follows an observation by Makumburage and Stapleton (2011) in a plant height uniformity study, where rating of an inbred in a single-stress environment was not predictive of the combined stress rating. Classification of germplasm into environmental response patterns is becoming a useful selection tool across a variety of traits (Winterhalter et al., 2011); use of these patterns as phenotypes for subsequent genetic mapping and selection will undoubtedly provide greater insight on the mechanisms through which biological networks within a given genotype interact with the environment.

Substantial progress has been made in understanding the physiological and genetic bases for the iron trait in cereals. Experimentation with mineral partitioning and accumulation both within a grain and along an inflorescence (Welch et al., 1993; Bänziger and Long, 2000; Calderini and Ortiz-Monasterio, 2003) has helped to delimit the physiological constraints of iron import and storage. Evaluation of specific combining ability and reciprocal effects on mineral density in rice has highlighted the importance of maternal parent selection (Gregorio et al., 2000). Exploration of genetic diversity in wild varieties and landraces has led to the identification of unexploited allelic variation that can be used in pre-breeding crosses (Chatzav et al., 2010). Alternative methods to screen mineral concentrations across germplasm with greater accuracy and speed (Baxter et al., 2013) or with lower cost and technical difficulty (Choi et al., 2007; Michenfelder, 2009) are currently being explored. The results reported here add to this body of knowledge, and stress the use of cross-environment comparisons to uncover emergent and novel phenotypic responses that could contribute to either adaptability or stability of grain Fe concentration, and better explain GxE effects. Further evaluation of the effect of nitrogen use efficiency (NUE) and water use efficiency (WUE) on kernel Fe concentration would be useful in determining the mechanism of mineral import under stress. Lastly, and perhaps most importantly, an optimization analysis of grain yield and micronutrient yield would be helpful in determining the bounds of the dilution effect on nutrient density.

## Author contributions

Ann E. Stapleton conceived and designed the experiments, Ann E. Stapleton, Abigail S. Michenfelder, and Catherine B. Kandianis performed the experiments, Ann E. Stapleton, Catherine B. Kandianis, Susan J. Simmons, and Abigail S. Michenfelder analyzed the data, Ann E. Stapleton, Catherine B. Kandianis, and Michael A. Grusak wrote the paper.

### Conflict of interest statement

The authors declare that the research was conducted in the absence of any commercial or financial relationships that could be construed as a potential conflict of interest.

## Supplementary material

Data Sheet 1 **Grain mineral and physiological data for each genotype- treatment combination is grouped into separate sheets in the xls file**. Inbred genotypes are listed by their letter-number codes using the maize genetics naming conventions. Mineral concentration data from ICP analysis includes experimental plot-specific entry numbers, the inbred ID, the ear number (as some measurements required kernels from more than one ear), while kernel and cob data are presented as entry means.

Data Sheet 2 **Input R code for mvpart R package and JMP scripts**.

Data Sheet 3 **Output of mvpart analysis**.

Data Sheet 4 **Effect estimates, LSmeans, and pairwise comparisons ANOVA output for iron concentration modeled with genotype[treatment] factors**.

Data Sheet 5 **Effect estimates, LSmeans, and pairwise comparisons ANOVA output for iron concentration modeled with genotype[treatment] and genotype^*^Mg factors**.

Data Sheet 6 **Correlation between kernel iron concentration and magnesium concentration**. Experiment-wide and treatment-wide correlations between kernel Fe and Mg concentration are indicated.

Presentation 1 **Partition analysis with three factors important for iron concentration (inbred, treatment, and Mg concentration)**.
