# Supplementary figures and images for: Abiotic stress growth conditions induce different responses in kernel iron concentration across genotypically distinct maize inbred varieties
Source: Front Plant Sci. 2013 Dec 4;4:488. doi: 10.3389/fpls.2013.00488 (PMC3850239; doi:10.3389/fpls.2013.00488)

inbreds with highest kernel iron

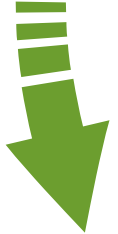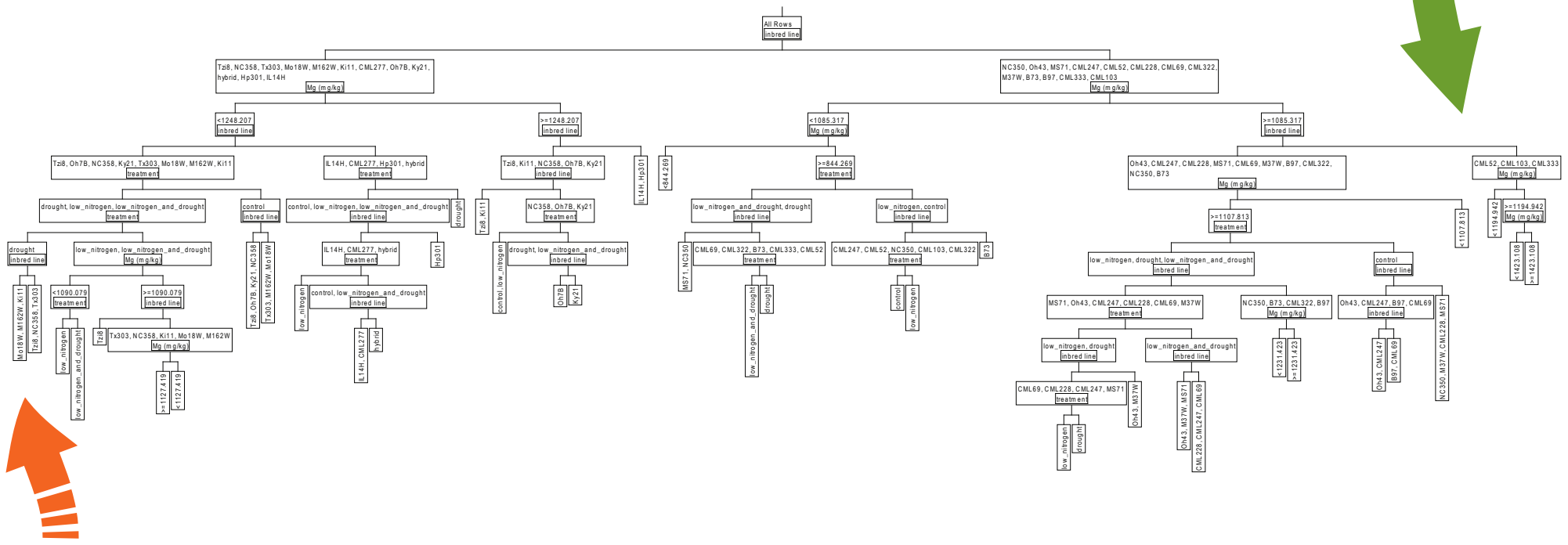

inbreds with lowest kernel iron content

Supplement: Data Sheet 1 — Grain mineral and physiological data for each genotype- treatment combination is grouped into separate sheets in the xls file. Inbred genotypes are listed by their letter-number codes using the maize genetics naming conventions. Mineral concentration data from ICP analysis includes experimental plot-specific entry numbers, the inbred ID, the ear number (as some measurements required kernels from more than one ear), while kernel and cob data are presented as entry means. [file DataSheet1.ZIP › 68761_Stapleton_Presentation_1.PDF]
