# Supplementary material for: Abiotic stress growth conditions induce different responses in kernel iron concentration across genotypically distinct maize inbred varieties
Source: Front Plant Sci. 2013 Dec 4;4:488. doi: 10.3389/fpls.2013.00488 (PMC3850239; doi:10.3389/fpls.2013.00488)
Supplement: Data Sheet 1 — Grain mineral and physiological data for each genotype- treatment combination is grouped into separate sheets in the xls file. Inbred genotypes are listed by their letter-number codes using the maize genetics naming conventions. Mineral concentration data from ICP analysis includes experimental plot-specific entry numbers, the inbred ID, the ear number (as some measurements required kernels from more than one ear), while kernel and cob data are presented as entry means. [file DataSheet1.ZIP › 68761_Stapleton_Data_Sheet_2.DOC]

**Datasheet 2 R code for MVPart analysis**

\## load software

library(mvpart)

###read in data

dat<-read.table('C:\\Documents and Settings\\User\\Desktop\\Work\\Ann\\iron_kwt_mvpart_2.csv',sep=',',header=TRUE)

###use Euclidean distances

fit1<-rpart(data.matrix(dat[,3:4])~treatment+inbred,dat,method='mrt',dissim='euc')

plot(fit1); text(fit1)

###use manhattan distances

fit2<-rpart(data.matrix(dat[,3:4])~treatment+inbred,dat,method='mrt',dissim='man')

plot(fit2); text(fit2)

####save predicted values from each fit

temp1<-predict(fit1)

temp2<-predict(fit2)

###see which fit is best (if what average absolute deviations take these numbers

\### and divide by 464

sum(abs(temp1-dat.temp))

[1] 840.0069

sum(abs(temp2-dat.temp))

[1] 914.516

> fit1

n= 232

node), split, n, deviance, yval

* denotes terminal node

1) root 232 10631.79000 10.354040

2) inbred=CML277,Hp301,hybrid,Ki11,Ky21,M162W,Mo18W,NC358,Oh7B,Tx303,Tzi8 97 2357.96500 8.224387

4) inbred=Ki11,M162W,Mo18W,NC358,Tx303,Tzi8 53	960.35500 7.390840

8) treatment=lowN,lowNlow,lowWate 38	527.55290 6.843553 *

9) treatment=control 15	272.55510 8.777300 *

5) inbred=CML277,Hp301,hybrid,Ky21,Oh7B 44 1066.20300 9.228432

10) treatment=control,lowN 19	286.41790 8.292947 *

11) treatment=lowNlow,lowWate 25	661.21190 9.939400 *

3) inbred=B73,B97,CML103,CML228,CML247,CML322,CML333,CML52,CML69,IL14H,M37W,MS71,NC350,Oh43 135 5242.23600 11.884240

6) inbred=CML228,CML247,CML322,CML52,CML69,IL14H,MS71,NC350,Oh43 73 2189.49300 10.785750

12) treatment=lowN,lowNlow,lowWate 55 1518.36700 10.255570

24) inbred=CML228,MS71,NC350 18	277.58700 9.226222 *

25) inbred=CML247,CML322,CML52,CML69,IL14H,Oh43 37 1125.74400 10.756340 *

13) treatment=control 18	419.05790 12.405750

26) inbred=CML322,CML52,CML69,IL14H,Oh43 12	140.96690 11.203000 *

27) inbred=CML228,MS71,NC350 6	71.81896 14.811250 *

7) inbred=B73,B97,CML103,CML333,M37W 62 2298.35600 13.177620

14) treatment=lowNlow,lowWate 33 1102.50900 12.032860

28) inbred=B73,B97,CML333,M37W 28	776.82900 11.564060 *

29) inbred=CML103 5	162.85100 14.658100 *

15) treatment=control,lowN 29	831.05630 14.480280

30) inbred=B73,B97,M37W 20	378.00830 13.714880 *

31) inbred=CML103,CML333 9	302.63920 16.181170 *
