# Supplementary material for: Abiotic stress growth conditions induce different responses in kernel iron concentration across genotypically distinct maize inbred varieties
Source: Front Plant Sci. 2013 Dec 4;4:488. doi: 10.3389/fpls.2013.00488 (PMC3850239; doi:10.3389/fpls.2013.00488)
Supplement: Data Sheet 1 — Grain mineral and physiological data for each genotype- treatment combination is grouped into separate sheets in the xls file. Inbred genotypes are listed by their letter-number codes using the maize genetics naming conventions. Mineral concentration data from ICP analysis includes experimental plot-specific entry numbers, the inbred ID, the ear number (as some measurements required kernels from more than one ear), while kernel and cob data are presented as entry means. [file DataSheet1.ZIP › 68761_Stapleton_Data_Sheet_4.DOC]

**Supplemental File 4 ANOVA details**

**Response Fe**

**Whole Model**

Summary of Fit

|  |  |
| --- | --- |
| RSquare | 0.635763 |
| RSquare Adj | 0.422925 |
| Root Mean Square Error | 5.139096 |
| Mean of Response | 20.20798 |
| Observations (or Sum Wgts) | 264 |

Analysis of Variance

| **Source** | **DF** | **Sum of Squares** | **Mean Square** | **F Ratio** |
| --- | --- | --- | --- | --- |
| Model | 97 | 7652.300 | 78.8897 | 2.9871 |
| Error | 166 | 4384.110 | 26.4103 | **Prob > F** |
| C. Total | 263 | 12036.411 |  | <.0001 |

Lack Of Fit

| **Source** | **DF** | **Sum of Squares** | **Mean Square** | **F Ratio** |
| --- | --- | --- | --- | --- |
| Lack Of Fit | 3 | 281.2463 | 93.7488 | 3.7245 |
| Pure Error | 163 | 4102.8641 | 25.1709 | **Prob > F** |
| Total Error | 166 | 4384.1103 |  | 0.0126 |
|  |  |  |  | **Max RSq** |
|  |  |  |  | 0.6591 |

Parameter Estimates

| **Term** |  | **Estimate** | **Std Error** | **t Ratio** | **Prob>\|t\|** |
| --- | --- | --- | --- | --- | --- |
| Intercept |  | 20.071807 | 0.331791 | 60.50 | <.0001 |
| treatment[control]:pedigree[B73] |  | 5.2848769 | 2.091368 | 2.53 | 0.0124 |
| treatment[control]:pedigree[B97] |  | 1.4708939 | 2.921232 | 0.50 | 0.6153 |
| treatment[control]:pedigree[CML103] |  | 17.353744 | 3.548156 | 4.89 | <.0001 |
| treatment[control]:pedigree[CML228] |  | 5.9152441 | 3.548156 | 1.67 | 0.0974 |
| treatment[control]:pedigree[CML247] |  | 0.7454687 | 2.542971 | 0.29 | 0.7698 |
| treatment[control]:pedigree[CML277] |  | -8.610756 | 3.548156 | -2.43 | 0.0163 |
| treatment[control]:pedigree[CML322] |  | 1.1887441 | 3.548156 | 0.34 | 0.7380 |
| treatment[control]:pedigree[CML333] |  | 7.7867441 | 3.548156 | 2.19 | 0.0296 |
| treatment[control]:pedigree[CML52] |  | 0.9337187 | 2.542971 | 0.37 | 0.7140 |
| treatment[control]:pedigree[CML69] |  | 0.8742441 | 3.548156 | 0.25 | 0.8057 |
| treatment[control]:pedigree[Hp301] |  | -6.594256 | 3.548156 | -1.86 | 0.0649 |
| treatment[control]:pedigree[hybrid] |  | -3.010756 | 3.548156 | -0.85 | 0.3974 |
| treatment[control]:pedigree[IL14H] |  | -1.524756 | 3.548156 | -0.43 | 0.6679 |
| treatment[control]:pedigree[Ki11] |  | -1.262756 | 3.548156 | -0.36 | 0.7224 |
| treatment[control]:pedigree[Ky21] |  | -6.293256 | 3.548156 | -1.77 | 0.0780 |
| treatment[control]:pedigree[M162W] |  | -3.568439 | 2.921232 | -1.22 | 0.2236 |
| treatment[control]:pedigree[M37W] |  | 5.7608939 | 2.921232 | 1.97 | 0.0503 |
| treatment[control]:pedigree[Mo18W] |  | -2.330439 | 2.921232 | -0.80 | 0.4261 |
| treatment[control]:pedigree[MS71] |  | 10.369744 | 3.548156 | 2.92 | 0.0040 |
| treatment[control]:pedigree[NC350] |  | 4.9592441 | 3.548156 | 1.40 | 0.1641 |
| treatment[control]:pedigree[NC358] |  | -6.291106 | 2.921232 | -2.15 | 0.0327 |
| treatment[control]:pedigree[Oh43] |  | -0.792256 | 3.548156 | -0.22 | 0.8236 |
| treatment[control]:pedigree[Oh7B] |  | -7.572773 | 2.921232 | -2.59 | 0.0104 |
| treatment[control]:pedigree[Tx303] |  | -4.465256 | 3.548156 | -1.26 | 0.2100 |
| treatment[lowN]:pedigree[B73] |  | 9.9404646 | 2.092395 | 4.75 | <.0001 |
| treatment[lowN]:pedigree[B97] |  | 8.310007 | 3.552593 | 2.34 | 0.0205 |
| treatment[lowN]:pedigree[CML103] |  | 6.9660691 | 2.923764 | 2.38 | 0.0183 |
| treatment[lowN]:pedigree[CML228] |  | -3.030493 | 3.552593 | -0.85 | 0.3949 |
| treatment[lowN]:pedigree[CML247] |  | -2.444993 | 3.552593 | -0.69 | 0.4923 |
| treatment[lowN]:pedigree[CML277] |  | -2.129264 | 2.923764 | -0.73 | 0.4675 |
| treatment[lowN]:pedigree[CML322] |  | 5.185007 | 3.552593 | 1.46 | 0.1463 |
| treatment[lowN]:pedigree[CML333] |  | 14.777007 | 3.552593 | 4.16 | <.0001 |
| treatment[lowN]:pedigree[CML52] |  | 2.2517358 | 2.923764 | 0.77 | 0.4423 |
| treatment[lowN]:pedigree[CML69] |  | -2.226493 | 3.552593 | -0.63 | 0.5317 |
| treatment[lowN]:pedigree[Hp301] |  | 3.529007 | 3.552593 | 0.99 | 0.3220 |
| treatment[lowN]:pedigree[hybrid] |  | -6.550931 | 2.923764 | -2.24 | 0.0264 |
| treatment[lowN]:pedigree[IL14H] |  | 0.8520691 | 2.923764 | 0.29 | 0.7711 |
| treatment[lowN]:pedigree[Ki11] |  | -6.322993 | 3.552593 | -1.78 | 0.0769 |
| treatment[lowN]:pedigree[Ky21] |  | -5.956493 | 3.552593 | -1.68 | 0.0955 |
| treatment[lowN]:pedigree[M162W] |  | -3.719931 | 2.923764 | -1.27 | 0.2050 |
| treatment[lowN]:pedigree[M37W] |  | 0.906007 | 3.552593 | 0.26 | 0.7990 |
| treatment[lowN]:pedigree[Mo18W] |  | -5.125931 | 2.923764 | -1.75 | 0.0814 |
| treatment[lowN]:pedigree[MS71] |  | -0.815264 | 2.923764 | -0.28 | 0.7807 |
| treatment[lowN]:pedigree[NC350] |  | -2.832264 | 2.923764 | -0.97 | 0.3341 |
| treatment[lowN]:pedigree[NC358] |  | -7.2264 | 2.544696 | -2.84 | 0.0051 |
| treatment[lowN]:pedigree[Oh43] |  | 6.709007 | 3.552593 | 1.89 | 0.0607 |
| treatment[lowN]:pedigree[Oh7B] |  | -2.682493 | 3.552593 | -0.76 | 0.4513 |
| treatment[lowN]:pedigree[P39] |  | 8.0808206 | 4.924628 | 1.64 | 0.1027 |
| treatment[lowN]:pedigree[Tx303] |  | -7.803264 | 2.923764 | -2.67 | 0.0084 |
| treatment[lowNlowWater]:pedigree[B73] |  | 1.2877331 | 1.820403 | 0.71 | 0.4803 |
| treatment[lowNlowWater]:pedigree[B97] |  | 7.7263525 | 3.544562 | 2.18 | 0.0307 |
| treatment[lowNlowWater]:pedigree[CML103] |  | 9.0273525 | 3.544562 | 2.55 | 0.0118 |
| treatment[lowNlowWater]:pedigree[CML228] |  | 4.6039661 | 2.918851 | 1.58 | 0.1166 |
| treatment[lowNlowWater]:pedigree[CML247] |  | 6.3253525 | 3.544562 | 1.78 | 0.0762 |
| treatment[lowNlowWater]:pedigree[CML277] |  | -2.665367 | 2.918851 | -0.91 | 0.3625 |
| treatment[lowNlowWater]:pedigree[CML322] |  | 0.6358525 | 3.544562 | 0.18 | 0.8579 |
| treatment[lowNlowWater]:pedigree[CML333] |  | 5.7443525 | 3.544562 | 1.62 | 0.1070 |
| treatment[lowNlowWater]:pedigree[CML52] |  | 2.7743525 | 3.544562 | 0.78 | 0.4349 |
| treatment[lowNlowWater]:pedigree[CML69] |  | 6.6262995 | 2.918851 | 2.27 | 0.0245 |
| treatment[lowNlowWater]:pedigree[Hp301] |  | -1.886147 | 3.544562 | -0.53 | 0.5954 |
| treatment[lowNlowWater]:pedigree[hybrid] |  | -1.119367 | 2.918851 | -0.38 | 0.7018 |
| treatment[lowNlowWater]:pedigree[IL14H] |  | 0.2822995 | 2.918851 | 0.10 | 0.9231 |
| treatment[lowNlowWater]:pedigree[Ki11] |  | -3.162367 | 2.918851 | -1.08 | 0.2802 |
| treatment[lowNlowWater]:pedigree[Ky21] |  | 2.1872995 | 2.918851 | 0.75 | 0.4547 |
| treatment[lowNlowWater]:pedigree[M162W] |  | -7.438147 | 3.544562 | -2.10 | 0.0374 |
| treatment[lowNlowWater]:pedigree[M37W] |  | -1.194147 | 3.544562 | -0.34 | 0.7366 |
| treatment[lowNlowWater]:pedigree[Mo18W] |  | -6.109647 | 3.544562 | -1.72 | 0.0866 |
| treatment[lowNlowWater]:pedigree[MS71] |  | -3.280034 | 2.918851 | -1.12 | 0.2627 |
| treatment[lowNlowWater]:pedigree[NC350] |  | 2.2158525 | 3.544562 | 0.63 | 0.5327 |
| treatment[lowNlowWater]:pedigree[NC358] |  | -4.300701 | 2.918851 | -1.47 | 0.1425 |
| treatment[lowNlowWater]:pedigree[Oh43] |  | -2.406147 | 3.544562 | -0.68 | 0.4982 |
| treatment[lowNlowWater]:pedigree[Oh7B] |  | -0.677977 | 2.541148 | -0.27 | 0.7900 |
| treatment[lowNlowWater]:pedigree[Tx303] |  | -6.460647 | 3.544562 | -1.82 | 0.0701 |
| treatment[lowWater]:pedigree[B73] |  | 2.6740747 | 2.090675 | 1.28 | 0.2027 |
| treatment[lowWater]:pedigree[B97] |  | 4.3372893 | 2.919953 | 1.49 | 0.1393 |
| treatment[lowWater]:pedigree[CML103] |  | 10.126956 | 2.919953 | 3.47 | 0.0007 |
| treatment[lowWater]:pedigree[CML228] |  | 0.7463373 | 3.546224 | 0.21 | 0.8336 |
| treatment[lowWater]:pedigree[CML247] |  | 0.1813373 | 3.546224 | 0.05 | 0.9593 |
| treatment[lowWater]:pedigree[CML277] |  | -0.816163 | 3.546224 | -0.23 | 0.8183 |
| treatment[lowWater]:pedigree[CML322] |  | 6.5238373 | 3.546224 | 1.84 | 0.0676 |
| treatment[lowWater]:pedigree[CML333] |  | 3.2083373 | 3.546224 | 0.90 | 0.3669 |
| treatment[lowWater]:pedigree[CML52] |  | 1.2222893 | 2.919953 | 0.42 | 0.6761 |
| treatment[lowWater]:pedigree[CML69] |  | 4.3706227 | 2.919953 | 1.50 | 0.1363 |
| treatment[lowWater]:pedigree[Hp301] |  | 0.0967653 | 2.541993 | 0.04 | 0.9697 |
| treatment[lowWater]:pedigree[hybrid] |  | 3.694956 | 2.919953 | 1.27 | 0.2075 |
| treatment[lowWater]:pedigree[IL14H] |  | -1.222377 | 2.919953 | -0.42 | 0.6760 |
| treatment[lowWater]:pedigree[Ki11] |  | -6.296377 | 2.919953 | -2.16 | 0.0325 |
| treatment[lowWater]:pedigree[Ky21] |  | 0.1208373 | 3.546224 | 0.03 | 0.9729 |
| treatment[lowWater]:pedigree[M162W] |  | -7.821163 | 3.546224 | -2.21 | 0.0288 |
| treatment[lowWater]:pedigree[M37W] |  | 7.5818373 | 3.546224 | 2.14 | 0.0340 |
| treatment[lowWater]:pedigree[Mo18W] |  | -11.31116 | 3.546224 | -3.19 | 0.0017 |
| treatment[lowWater]:pedigree[MS71] |  | 0.534956 | 2.919953 | 0.18 | 0.8549 |
| treatment[lowWater]:pedigree[NC350] |  | -1.692663 | 3.546224 | -0.48 | 0.6338 |
| treatment[lowWater]:pedigree[NC358] |  | -6.165663 | 3.546224 | -1.74 | 0.0839 |
| treatment[lowWater]:pedigree[Oh43] |  | -1.078163 | 3.546224 | -0.30 | 0.7615 |
| treatment[lowWater]:pedigree[Oh7B] |  | 1.1966227 | 2.919953 | 0.41 | 0.6825 |
| treatment[lowWater]:pedigree[Tx303] |  | -4.065663 | 3.546224 | -1.15 | 0.2532 |

Effect Tests

| **Source** | **Nparm** | **DF** | **Sum of Squares** | **F Ratio** | **Prob > F** |  |
| --- | --- | --- | --- | --- | --- | --- |
| pedigree[treatment] | 97 | 97 | 7652.3004 | 2.9871 | <.0001 |  |

Sorted Parameter Estimates

| **Term** |  | **Estimate** | **Std Error** | **t Ratio** | **t Ratio** | **Prob>\|t\|** |
| --- | --- | --- | --- | --- | --- | --- |
| treatment[control]:pedigree[CML103] |  | 17.353744 | 3.548156 | 4.89 |  | <.0001 |
| treatment[lowN]:pedigree[B73] |  | 9.9404646 | 2.092395 | 4.75 |  | <.0001 |
| treatment[lowN]:pedigree[CML333] |  | 14.777007 | 3.552593 | 4.16 |  | <.0001 |
| treatment[lowWater]:pedigree[CML103] |  | 10.126956 | 2.919953 | 3.47 |  | 0.0007 |
| treatment[lowWater]:pedigree[Mo18W] |  | -11.31116 | 3.546224 | -3.19 |  | 0.0017 |
| treatment[control]:pedigree[MS71] |  | 10.369744 | 3.548156 | 2.92 |  | 0.0040 |
| treatment[lowN]:pedigree[NC358] |  | -7.2264 | 2.544696 | -2.84 |  | 0.0051 |
| treatment[lowN]:pedigree[Tx303] |  | -7.803264 | 2.923764 | -2.67 |  | 0.0084 |
| treatment[control]:pedigree[Oh7B] |  | -7.572773 | 2.921232 | -2.59 |  | 0.0104 |
| treatment[lowNlowWater]:pedigree[CML103] |  | 9.0273525 | 3.544562 | 2.55 |  | 0.0118 |
| treatment[control]:pedigree[B73] |  | 5.2848769 | 2.091368 | 2.53 |  | 0.0124 |
| treatment[control]:pedigree[CML277] |  | -8.610756 | 3.548156 | -2.43 |  | 0.0163 |
| treatment[lowN]:pedigree[CML103] |  | 6.9660691 | 2.923764 | 2.38 |  | 0.0183 |
| treatment[lowN]:pedigree[B97] |  | 8.310007 | 3.552593 | 2.34 |  | 0.0205 |
| treatment[lowNlowWater]:pedigree[CML69] |  | 6.6262995 | 2.918851 | 2.27 |  | 0.0245 |
| treatment[lowN]:pedigree[hybrid] |  | -6.550931 | 2.923764 | -2.24 |  | 0.0264 |
| treatment[lowWater]:pedigree[M162W] |  | -7.821163 | 3.546224 | -2.21 |  | 0.0288 |
| treatment[control]:pedigree[CML333] |  | 7.7867441 | 3.548156 | 2.19 |  | 0.0296 |
| treatment[lowNlowWater]:pedigree[B97] |  | 7.7263525 | 3.544562 | 2.18 |  | 0.0307 |
| treatment[lowWater]:pedigree[Ki11] |  | -6.296377 | 2.919953 | -2.16 |  | 0.0325 |
| treatment[control]:pedigree[NC358] |  | -6.291106 | 2.921232 | -2.15 |  | 0.0327 |
| treatment[lowWater]:pedigree[M37W] |  | 7.5818373 | 3.546224 | 2.14 |  | 0.0340 |
| treatment[lowNlowWater]:pedigree[M162W] |  | -7.438147 | 3.544562 | -2.10 |  | 0.0374 |
| treatment[control]:pedigree[M37W] |  | 5.7608939 | 2.921232 | 1.97 |  | 0.0503 |
| treatment[lowN]:pedigree[Oh43] |  | 6.709007 | 3.552593 | 1.89 |  | 0.0607 |
| treatment[control]:pedigree[Hp301] |  | -6.594256 | 3.548156 | -1.86 |  | 0.0649 |
| treatment[lowWater]:pedigree[CML322] |  | 6.5238373 | 3.546224 | 1.84 |  | 0.0676 |
| treatment[lowNlowWater]:pedigree[Tx303] |  | -6.460647 | 3.544562 | -1.82 |  | 0.0701 |
| treatment[lowNlowWater]:pedigree[CML247] |  | 6.3253525 | 3.544562 | 1.78 |  | 0.0762 |
| treatment[lowN]:pedigree[Ki11] |  | -6.322993 | 3.552593 | -1.78 |  | 0.0769 |
| treatment[control]:pedigree[Ky21] |  | -6.293256 | 3.548156 | -1.77 |  | 0.0780 |
| treatment[lowN]:pedigree[Mo18W] |  | -5.125931 | 2.923764 | -1.75 |  | 0.0814 |
| treatment[lowWater]:pedigree[NC358] |  | -6.165663 | 3.546224 | -1.74 |  | 0.0839 |
| treatment[lowNlowWater]:pedigree[Mo18W] |  | -6.109647 | 3.544562 | -1.72 |  | 0.0866 |
| treatment[lowN]:pedigree[Ky21] |  | -5.956493 | 3.552593 | -1.68 |  | 0.0955 |
| treatment[control]:pedigree[CML228] |  | 5.9152441 | 3.548156 | 1.67 |  | 0.0974 |
| treatment[lowN]:pedigree[P39] |  | 8.0808206 | 4.924628 | 1.64 |  | 0.1027 |
| treatment[lowNlowWater]:pedigree[CML333] |  | 5.7443525 | 3.544562 | 1.62 |  | 0.1070 |
| treatment[lowNlowWater]:pedigree[CML228] |  | 4.6039661 | 2.918851 | 1.58 |  | 0.1166 |
| treatment[lowWater]:pedigree[CML69] |  | 4.3706227 | 2.919953 | 1.50 |  | 0.1363 |
| treatment[lowWater]:pedigree[B97] |  | 4.3372893 | 2.919953 | 1.49 |  | 0.1393 |
| treatment[lowNlowWater]:pedigree[NC358] |  | -4.300701 | 2.918851 | -1.47 |  | 0.1425 |
| treatment[lowN]:pedigree[CML322] |  | 5.185007 | 3.552593 | 1.46 |  | 0.1463 |
| treatment[control]:pedigree[NC350] |  | 4.9592441 | 3.548156 | 1.40 |  | 0.1641 |
| treatment[lowWater]:pedigree[B73] |  | 2.6740747 | 2.090675 | 1.28 |  | 0.2027 |
| treatment[lowN]:pedigree[M162W] |  | -3.719931 | 2.923764 | -1.27 |  | 0.2050 |
| treatment[lowWater]:pedigree[hybrid] |  | 3.694956 | 2.919953 | 1.27 |  | 0.2075 |
| treatment[control]:pedigree[Tx303] |  | -4.465256 | 3.548156 | -1.26 |  | 0.2100 |
| treatment[control]:pedigree[M162W] |  | -3.568439 | 2.921232 | -1.22 |  | 0.2236 |
| treatment[lowWater]:pedigree[Tx303] |  | -4.065663 | 3.546224 | -1.15 |  | 0.2532 |
| treatment[lowNlowWater]:pedigree[MS71] |  | -3.280034 | 2.918851 | -1.12 |  | 0.2627 |
| treatment[lowNlowWater]:pedigree[Ki11] |  | -3.162367 | 2.918851 | -1.08 |  | 0.2802 |
| treatment[lowN]:pedigree[Hp301] |  | 3.529007 | 3.552593 | 0.99 |  | 0.3220 |
| treatment[lowN]:pedigree[NC350] |  | -2.832264 | 2.923764 | -0.97 |  | 0.3341 |
| treatment[lowNlowWater]:pedigree[CML277] |  | -2.665367 | 2.918851 | -0.91 |  | 0.3625 |
| treatment[lowWater]:pedigree[CML333] |  | 3.2083373 | 3.546224 | 0.90 |  | 0.3669 |
| treatment[lowN]:pedigree[CML228] |  | -3.030493 | 3.552593 | -0.85 |  | 0.3949 |
| treatment[control]:pedigree[hybrid] |  | -3.010756 | 3.548156 | -0.85 |  | 0.3974 |
| treatment[control]:pedigree[Mo18W] |  | -2.330439 | 2.921232 | -0.80 |  | 0.4261 |
| treatment[lowNlowWater]:pedigree[CML52] |  | 2.7743525 | 3.544562 | 0.78 |  | 0.4349 |
| treatment[lowN]:pedigree[CML52] |  | 2.2517358 | 2.923764 | 0.77 |  | 0.4423 |
| treatment[lowN]:pedigree[Oh7B] |  | -2.682493 | 3.552593 | -0.76 |  | 0.4513 |
| treatment[lowNlowWater]:pedigree[Ky21] |  | 2.1872995 | 2.918851 | 0.75 |  | 0.4547 |
| treatment[lowN]:pedigree[CML277] |  | -2.129264 | 2.923764 | -0.73 |  | 0.4675 |
| treatment[lowNlowWater]:pedigree[B73] |  | 1.2877331 | 1.820403 | 0.71 |  | 0.4803 |
| treatment[lowN]:pedigree[CML247] |  | -2.444993 | 3.552593 | -0.69 |  | 0.4923 |
| treatment[lowNlowWater]:pedigree[Oh43] |  | -2.406147 | 3.544562 | -0.68 |  | 0.4982 |
| treatment[lowN]:pedigree[CML69] |  | -2.226493 | 3.552593 | -0.63 |  | 0.5317 |
| treatment[lowNlowWater]:pedigree[NC350] |  | 2.2158525 | 3.544562 | 0.63 |  | 0.5327 |
| treatment[lowNlowWater]:pedigree[Hp301] |  | -1.886147 | 3.544562 | -0.53 |  | 0.5954 |
| treatment[control]:pedigree[B97] |  | 1.4708939 | 2.921232 | 0.50 |  | 0.6153 |
| treatment[lowWater]:pedigree[NC350] |  | -1.692663 | 3.546224 | -0.48 |  | 0.6338 |
| treatment[control]:pedigree[IL14H] |  | -1.524756 | 3.548156 | -0.43 |  | 0.6679 |
| treatment[lowWater]:pedigree[IL14H] |  | -1.222377 | 2.919953 | -0.42 |  | 0.6760 |
| treatment[lowWater]:pedigree[CML52] |  | 1.2222893 | 2.919953 | 0.42 |  | 0.6761 |
| treatment[lowWater]:pedigree[Oh7B] |  | 1.1966227 | 2.919953 | 0.41 |  | 0.6825 |
| treatment[lowNlowWater]:pedigree[hybrid] |  | -1.119367 | 2.918851 | -0.38 |  | 0.7018 |
| treatment[control]:pedigree[CML52] |  | 0.9337187 | 2.542971 | 0.37 |  | 0.7140 |
| treatment[control]:pedigree[Ki11] |  | -1.262756 | 3.548156 | -0.36 |  | 0.7224 |
| treatment[lowNlowWater]:pedigree[M37W] |  | -1.194147 | 3.544562 | -0.34 |  | 0.7366 |
| treatment[control]:pedigree[CML322] |  | 1.1887441 | 3.548156 | 0.34 |  | 0.7380 |
| treatment[lowWater]:pedigree[Oh43] |  | -1.078163 | 3.546224 | -0.30 |  | 0.7615 |
| treatment[control]:pedigree[CML247] |  | 0.7454687 | 2.542971 | 0.29 |  | 0.7698 |
| treatment[lowN]:pedigree[IL14H] |  | 0.8520691 | 2.923764 | 0.29 |  | 0.7711 |
| treatment[lowN]:pedigree[MS71] |  | -0.815264 | 2.923764 | -0.28 |  | 0.7807 |
| treatment[lowNlowWater]:pedigree[Oh7B] |  | -0.677977 | 2.541148 | -0.27 |  | 0.7900 |
| treatment[lowN]:pedigree[M37W] |  | 0.906007 | 3.552593 | 0.26 |  | 0.7990 |
| treatment[control]:pedigree[CML69] |  | 0.8742441 | 3.548156 | 0.25 |  | 0.8057 |
| treatment[lowWater]:pedigree[CML277] |  | -0.816163 | 3.546224 | -0.23 |  | 0.8183 |
| treatment[control]:pedigree[Oh43] |  | -0.792256 | 3.548156 | -0.22 |  | 0.8236 |
| treatment[lowWater]:pedigree[CML228] |  | 0.7463373 | 3.546224 | 0.21 |  | 0.8336 |
| treatment[lowWater]:pedigree[MS71] |  | 0.534956 | 2.919953 | 0.18 |  | 0.8549 |
| treatment[lowNlowWater]:pedigree[CML322] |  | 0.6358525 | 3.544562 | 0.18 |  | 0.8579 |
| treatment[lowNlowWater]:pedigree[IL14H] |  | 0.2822995 | 2.918851 | 0.10 |  | 0.9231 |
| treatment[lowWater]:pedigree[CML247] |  | 0.1813373 | 3.546224 | 0.05 |  | 0.9593 |
| treatment[lowWater]:pedigree[Hp301] |  | 0.0967653 | 2.541993 | 0.04 |  | 0.9697 |
| treatment[lowWater]:pedigree[Ky21] |  | 0.1208373 | 3.546224 | 0.03 |  | 0.9729 |

**pedigree[treatment]**

**Slice treatment=control**

|  |  |
| --- | --- |
| Sum of Squares | 2281.0402904 |
| Numerator DF | 24 |
| Denominator DF | 166 |
| F Ratio | 3.5987222301 |
| Prob > F | 5.4698247e-7 |

**Slice treatment=lowN**

|  |  |
| --- | --- |
| Sum of Squares | 2448.9233298 |
| Numerator DF | 24 |
| Denominator DF | 166 |
| F Ratio | 3.8635857788 |
| Prob > F | 1.1286055e-7 |

**Slice treatment=lowNlowWater**

|  |  |
| --- | --- |
| Sum of Squares | 1341.5287709 |
| Numerator DF | 24 |
| Denominator DF | 166 |
| F Ratio | 2.1164858115 |
| Prob > F | 0.0032096828 |

**Slice treatment=lowWater**

|  |  |
| --- | --- |
| Sum of Squares | 1429.1370017 |
| Numerator DF | 24 |
| Denominator DF | 166 |
| F Ratio | 2.2547024353 |
| Prob > F | 0.0014881545 |

**Slice pedigree=B73**

|  |  |
| --- | --- |
| Sum of Squares | 260.58287801 |
| Numerator DF | 2 |
| Denominator DF | 166 |
| F Ratio | 4.9333564156 |
| Prob > F | 0.0082933248 |

**Slice pedigree=B97**

|  |  |
| --- | --- |
| Sum of Squares | 76.627502225 |
| Numerator DF | 2 |
| Denominator DF | 166 |
| F Ratio | 1.4507122747 |
| Prob > F | 0.2373593634 |

**Slice pedigree=CML103**

|  |  |
| --- | --- |
| Sum of Squares | 141.64484575 |
| Numerator DF | 2 |
| Denominator DF | 166 |
| F Ratio | 2.6816209639 |
| Prob > F | 0.0714175123 |

**Slice pedigree=CML228**

|  |  |
| --- | --- |
| Sum of Squares | 101.59385876 |
| Numerator DF | 2 |
| Denominator DF | 166 |
| F Ratio | 1.9233754678 |
| Prob > F | 0.1493551138 |

**Slice pedigree=CML247**

|  |  |
| --- | --- |
| Sum of Squares | 83.679303976 |
| Numerator DF | 2 |
| Denominator DF | 166 |
| F Ratio | 1.5842170225 |
| Prob > F | 0.2081934141 |

**Slice pedigree=CML277**

|  |  |
| --- | --- |
| Sum of Squares | 60.88770663 |
| Numerator DF | 2 |
| Denominator DF | 166 |
| F Ratio | 1.1527263818 |
| Prob > F | 0.318289146 |

**Slice pedigree=CML322**

|  |  |
| --- | --- |
| Sum of Squares | 25.828926435 |
| Numerator DF | 2 |
| Denominator DF | 166 |
| F Ratio | 0.488993371 |
| Prob > F | 0.6141239153 |

**Slice pedigree=CML333**

|  |  |
| --- | --- |
| Sum of Squares | 94.072471634 |
| Numerator DF | 2 |
| Denominator DF | 166 |
| F Ratio | 1.7809805272 |
| Prob > F | 0.1716767794 |

**Slice pedigree=CML52**

|  |  |
| --- | --- |
| Sum of Squares | 5.689609129 |
| Numerator DF | 2 |
| Denominator DF | 166 |
| F Ratio | 0.1077157099 |
| Prob > F | 0.8979455254 |

**Slice pedigree=CML69**

|  |  |
| --- | --- |
| Sum of Squares | 104.98854433 |
| Numerator DF | 2 |
| Denominator DF | 166 |
| F Ratio | 1.9876436729 |
| Prob > F | 0.1402656191 |

**Slice pedigree=Hp301**

|  |  |
| --- | --- |
| Sum of Squares | 107.5520757 |
| Numerator DF | 2 |
| Denominator DF | 166 |
| F Ratio | 2.0361764621 |
| Prob > F | 0.1337742532 |

**Slice pedigree=hybrid**

|  |  |
| --- | --- |
| Sum of Squares | 46.726295477 |
| Numerator DF | 2 |
| Denominator DF | 166 |
| F Ratio | 0.8846224715 |
| Prob > F | 0.4148071756 |

**Slice pedigree=IL14H**

|  |  |
| --- | --- |
| Sum of Squares | 7.351712173 |
| Numerator DF | 2 |
| Denominator DF | 166 |
| F Ratio | 0.1391826535 |
| Prob > F | 0.8701705204 |

**Slice pedigree=Ki11**

|  |  |
| --- | --- |
| Sum of Squares | 27.532081689 |
| Numerator DF | 2 |
| Denominator DF | 166 |
| F Ratio | 0.5212375152 |
| Prob > F | 0.5947538495 |

**Slice pedigree=Ky21**

|  |  |
| --- | --- |
| Sum of Squares | 123.16628599 |
| Numerator DF | 2 |
| Denominator DF | 166 |
| F Ratio | 2.3317847734 |
| Prob > F | 0.1002958205 |

**Slice pedigree=M162W**

|  |  |
| --- | --- |
| Sum of Squares | 22.599709456 |
| Numerator DF | 2 |
| Denominator DF | 166 |
| F Ratio | 0.4278578182 |
| Prob > F | 0.6526209399 |

**Slice pedigree=M37W**

|  |  |
| --- | --- |
| Sum of Squares | 66.724796667 |
| Numerator DF | 2 |
| Denominator DF | 166 |
| F Ratio | 1.2632342011 |
| Prob > F | 0.2854416574 |

**Slice pedigree=Mo18W**

|  |  |
| --- | --- |
| Sum of Squares | 20.989635414 |
| Numerator DF | 2 |
| Denominator DF | 166 |
| F Ratio | 0.3973758879 |
| Prob > F | 0.6727189376 |

**Slice pedigree=MS71**

|  |  |
| --- | --- |
| Sum of Squares | 250.93009936 |
| Numerator DF | 2 |
| Denominator DF | 166 |
| F Ratio | 4.7506099594 |
| Prob > F | 0.0098564318 |

**Slice pedigree=NC350**

|  |  |
| --- | --- |
| Sum of Squares | 81.180033224 |
| Numerator DF | 2 |
| Denominator DF | 166 |
| F Ratio | 1.5369008155 |
| Prob > F | 0.2180905873 |

**Slice pedigree=NC358**

|  |  |
| --- | --- |
| Sum of Squares | 15.324167401 |
| Numerator DF | 2 |
| Denominator DF | 166 |
| F Ratio | 0.2901172177 |
| Prob > F | 0.7485544292 |

**Slice pedigree=Oh43**

|  |  |
| --- | --- |
| Sum of Squares | 99.188056846 |
| Numerator DF | 2 |
| Denominator DF | 166 |
| F Ratio | 1.8778288133 |
| Prob > F | 0.156155737 |

**Slice pedigree=Oh7B**

|  |  |
| --- | --- |
| Sum of Squares | 85.365846399 |
| Numerator DF | 2 |
| Denominator DF | 166 |
| F Ratio | 1.6161466525 |
| Prob > F | 0.2017727028 |

**Slice pedigree=P39**

WARNING: Non-Testable Contrast

|  |  |
| --- | --- |
| Sum of Squares | 849.14480016 |
| Numerator DF | 1 |
| Denominator DF | 166 |
| F Ratio | 32.152027636 |
| Prob > F | 6.1910047e-8 |

**Slice pedigree=Tx303**

|  |  |
| --- | --- |
| Sum of Squares | 13.915972177 |
| Numerator DF | 2 |
| Denominator DF | 166 |
| F Ratio | 0.2634572584 |
| Prob > F | 0.7687111404 |

**Slice pedigree=Tzi8**

|  |  |
| --- | --- |
| Sum of Squares | 3.9777757431 |
| Numerator DF | 2 |
| Denominator DF | 166 |
| F Ratio | 0.0753072713 |
| Prob > F | 0.9274901281 |

**LSMeans Differences Tukey HSD**

α=

0.050	Q=

4.40465

| **Level** |  |  |  |  |  | **Least Sq Mean** |
| --- | --- | --- | --- | --- | --- | --- |
| [control]CML103 | A |  |  |  |  | 37.425551 |
| [lowN]CML333 | A | B |  |  |  | 34.848814 |
| [control]MS71 | A | B | C | D | E | 30.441551 |
| [lowWater]CML103 | A | B | C |  | E | 30.198763 |
| [lowN]B73 | A | B |  |  | E | 30.012271 |
| [lowNlowWater]CML103 | A | B | C | D | E | 29.099159 |
| [lowN]B97 | A | B | C | D | E | 28.381814 |
| [lowN]P39 | A | B | C | D | E | 28.152627 |
| [control]CML333 | A | B | C | D | E | 27.858551 |
| [lowNlowWater]B97 | A | B | C | D | E | 27.798159 |
| [lowWater]M37W | A | B | C | D | E | 27.653644 |
| [lowN]CML103 | A | B | C | D | E | 27.037876 |
| [lowN]Oh43 | A | B | C | D | E | 26.780814 |
| [lowNlowWater]CML69 | A | B | C | D | E | 26.698106 |
| [lowWater]CML322 | A | B | C | D | E | 26.595644 |
| [lowNlowWater]CML247 | A | B | C | D | E | 26.397159 |
| [control]CML228 | A | B | C | D | E | 25.987051 |
| [control]M37W | A | B | C | D | E | 25.832700 |
| [lowNlowWater]CML333 | A | B | C | D | E | 25.816159 |
| [control]B73 | A | B | C | D | E | 25.356684 |
| [lowN]CML322 | A | B | C | D | E | 25.256814 |
| [control]NC350 | A | B | C | D | E | 25.031051 |
| [lowNlowWater]CML228 | A | B | C | D | E | 24.675773 |
| [lowWater]CML69 | A | B | C | D | E | 24.442429 |
| [lowWater]B97 | A | B | C | D | E | 24.409096 |
| [lowWater]hybrid | A | B | C | D | E | 23.766763 |
| [lowN]Hp301 | A | B | C | D | E | 23.600814 |
| [lowWater]CML333 | A | B | C | D | E | 23.280144 |
| [lowNlowWater]CML52 | A | B | C | D | E | 22.846159 |
| [lowWater]B73 | A | B | C | D | E | 22.745881 |
| [lowN]CML52 | A | B | C | D | E | 22.323542 |
| [lowNlowWater]NC350 | A | B | C | D | E | 22.287659 |
| [lowNlowWater]Ky21 | A | B | C | D | E | 22.259106 |
| [control]B97 | A | B | C | D | E | 21.542700 |
| [lowNlowWater]B73 | A | B | C | D | E | 21.359540 |
| [lowWater]CML52 | A | B | C | D | E | 21.294096 |
| [lowWater]Oh7B | A | B | C | D | E | 21.268429 |
| [control]CML322 | A | B | C | D | E | 21.260551 |
| [control]CML52 | A | B | C | D | E | 21.005525 |
| [lowN]M37W | A | B | C | D | E | 20.977814 |
| [control]CML69 | A | B | C | D | E | 20.946051 |
| [lowN]IL14H | A | B | C | D | E | 20.923876 |
| [lowWater]CML228 | A | B | C | D | E | 20.818144 |
| [control]CML247 | A | B | C | D | E | 20.817275 |
| [lowNlowWater]CML322 | A | B | C | D | E | 20.707659 |
| [lowWater]MS71 | A | B | C | D | E | 20.606763 |
| [lowNlowWater]IL14H | A | B | C | D | E | 20.354106 |
| [lowWater]CML247 | A | B | C | D | E | 20.253144 |
| [lowWater]Ky21 | A | B | C | D | E | 20.192644 |
| [lowWater]Hp301 | A | B | C | D | E | 20.168572 |
| [lowNlowWater]Oh7B | A | B | C | D | E | 19.393830 |
| [control]Oh43 | A | B | C | D | E | 19.279551 |
| [lowN]MS71 | A | B | C | D | E | 19.256542 |
| [lowWater]CML277 | A | B | C | D | E | 19.255644 |
| [lowWater]Oh43 | A | B | C | D | E | 18.993644 |
| [lowNlowWater]hybrid | A | B | C | D | E | 18.952439 |
| [lowNlowWater]M37W | A | B | C | D | E | 18.877659 |
| [lowWater]IL14H | A | B | C | D | E | 18.849429 |
| [control]Ki11 | A | B | C | D | E | 18.809051 |
| [control]IL14H | A | B | C | D | E | 18.547051 |
| [lowWater]NC350 | A | B | C | D | E | 18.379144 |
| [lowNlowWater]Hp301 | A | B | C | D | E | 18.185659 |
| [lowN]CML277 | A | B | C | D | E | 17.942542 |
| [lowN]CML69 | A | B | C | D | E | 17.845314 |
| [control]Mo18W | A | B | C | D | E | 17.741367 |
| [lowNlowWater]Oh43 | A | B | C | D | E | 17.665659 |
| [lowN]CML247 | A | B | C | D | E | 17.626814 |
| [lowNlowWater]CML277 | A | B | C | D | E | 17.406439 |
| [lowN]Oh7B | A | B | C | D | E | 17.389314 |
| [lowN]NC350 | A | B | C | D | E | 17.239542 |
| [control]hybrid | A | B | C | D | E | 17.061051 |
| [lowN]CML228 | A | B | C | D | E | 17.041314 |
| [lowNlowWater]Ki11 |  | B | C | D | E | 16.909439 |
| [lowNlowWater]MS71 |  | B | C | D | E | 16.791773 |
| [control]M162W |  | B | C | D | E | 16.503367 |
| [lowN]M162W |  | B | C | D | E | 16.351876 |
| [lowWater]Tx303 | A | B | C | D | E | 16.006144 |
| [lowNlowWater]NC358 |  | B | C | D | E | 15.771106 |
| [control]Tx303 | A | B | C | D | E | 15.606551 |
| [lowN]Mo18W |  | B | C | D | E | 14.945876 |
| [lowN]Ky21 |  | B | C | D | E | 14.115314 |
| [lowNlowWater]Mo18W |  | B | C | D | E | 13.962159 |
| [lowWater]Tzi8 |  | B | C | D | E | 13.924144 |
| [lowWater]NC358 |  | B | C | D | E | 13.906144 |
| [control]NC358 |  |  | C | D |  | 13.780700 |
| [control]Ky21 |  | B | C | D | E | 13.778551 |
| [lowWater]Ki11 |  |  | C | D |  | 13.775429 |
| [lowN]Ki11 |  | B | C | D | E | 13.748814 |
| [lowNlowWater]Tx303 |  | B | C | D | E | 13.611159 |
| [lowN]hybrid |  |  | C | D |  | 13.520876 |
| [control]Hp301 |  | B | C | D | E | 13.477551 |
| [lowN]NC358 |  |  |  | D |  | 12.845407 |
| [lowNlowWater]M162W |  |  | C | D | E | 12.633659 |
| [control]Oh7B |  |  | C | D |  | 12.499034 |
| [lowN]Tx303 |  |  | C | D |  | 12.268542 |
| [lowWater]M162W |  |  | C | D | E | 12.250644 |
| [control]CML277 |  |  | C | D |  | 11.461051 |
| [lowN]Tzi8 |  |  | C | D |  | 11.431814 |
| [lowNlowWater]Tzi8 |  |  |  | D |  | 11.335439 |
| [control]Tzi8 |  |  |  | D |  | 9.745051 |
| [lowWater]Mo18W |  |  |  | D |  | 8.760644 |

Levels not connected by same letter are significantly different.
