# Supplementary material for: Abiotic stress growth conditions induce different responses in kernel iron concentration across genotypically distinct maize inbred varieties
Source: Front Plant Sci. 2013 Dec 4;4:488. doi: 10.3389/fpls.2013.00488 (PMC3850239; doi:10.3389/fpls.2013.00488)
Supplement: Data Sheet 1 — Grain mineral and physiological data for each genotype- treatment combination is grouped into separate sheets in the xls file. Inbred genotypes are listed by their letter-number codes using the maize genetics naming conventions. Mineral concentration data from ICP analysis includes experimental plot-specific entry numbers, the inbred ID, the ear number (as some measurements required kernels from more than one ear), while kernel and cob data are presented as entry means. [file DataSheet1.ZIP › 68761_Stapleton_Data_Sheet_5.DOC]

**Supplemental File 5**

**Response Fe**

**Whole Model**

Summary of Fit

|  |  |
| --- | --- |
| RSquare | 0.725006 |
| RSquare Adj | 0.487067 |
| Root Mean Square Error | 4.845079 |
| Mean of Response | 20.20798 |
| Observations (or Sum Wgts) | 264 |

Analysis of Variance

| **Source** | **DF** | **Sum of Squares** | **Mean Square** | **F Ratio** |
| --- | --- | --- | --- | --- |
| Model | 122 | 8726.465 | 71.5284 | 3.0470 |
| Error | 141 | 3309.945 | 23.4748 | **Prob > F** |
| C. Total | 263 | 12036.411 |  | <.0001 |

Effect Tests

| **Source** | **Nparm** | **DF** | **Sum of Squares** | **F Ratio** | **Prob > F** |  |
| --- | --- | --- | --- | --- | --- | --- |
| pedigree*Mg | 25 | 25 | 1074.1650 | 1.8303 | 0.0150 |  |
| pedigree[treatment] | 97 | 97 | 6213.4730 | 2.7287 | <.0001 |  |

Sorted Parameter Estimates

| **Term** |  | **Estimate** | **Std Error** | **t Ratio** | **t Ratio** | **Prob>\|t\|** |
| --- | --- | --- | --- | --- | --- | --- |
| treatment[lowN]:pedigree[B73] |  | 11.738592 | 2.291868 | 5.12 |  | <.0001 |
| treatment[control]:pedigree[B73] |  | 10.478818 | 2.86744 | 3.65 |  | 0.0004 |
| treatment[lowWater]:pedigree[Mo18W] |  | -12.01313 | 3.433132 | -3.50 |  | 0.0006 |
| treatment[lowN]:pedigree[P39] |  | 56.618603 | 16.23645 | 3.49 |  | 0.0007 |
| pedigree[P39]*(Mg-1171.6) |  | -0.3044 | 0.097689 | -3.12 |  | 0.0022 |
| pedigree[CML52]*(Mg-1171.6) |  | 0.0645317 | 0.02377 | 2.71 |  | 0.0075 |
| treatment[control]:pedigree[CML103] |  | 11.879765 | 4.379328 | 2.71 |  | 0.0075 |
| treatment[lowWater]:pedigree[CML52] |  | 20.010824 | 7.453904 | 2.68 |  | 0.0081 |
| treatment[lowN]:pedigree[NC358] |  | -6.976253 | 2.610067 | -2.67 |  | 0.0084 |
| treatment[lowNlowWater]:pedigree[CML103] |  | 9.1025772 | 3.423049 | 2.66 |  | 0.0087 |
| treatment[lowNlowWater]:pedigree[CML52] |  | 13.031642 | 4.932453 | 2.64 |  | 0.0092 |
| treatment[control]:pedigree[CML52] |  | 12.275593 | 4.663405 | 2.63 |  | 0.0094 |
| pedigree[B73]*(Mg-1171.6) |  | 0.0227265 | 0.00872 | 2.61 |  | 0.0101 |
| treatment[lowWater]:pedigree[CML103] |  | 7.6014302 | 2.960009 | 2.57 |  | 0.0113 |
| treatment[control]:pedigree[Oh7B] |  | -7.41587 | 2.904744 | -2.55 |  | 0.0117 |
| treatment[lowN]:pedigree[CML52] |  | 11.283377 | 4.497501 | 2.51 |  | 0.0132 |
| treatment[control]:pedigree[CML333] |  | 8.0946219 | 3.43677 | 2.36 |  | 0.0199 |
| treatment[control]:pedigree[CML277] |  | -16.6722 | 7.151504 | -2.33 |  | 0.0212 |
| treatment[lowNlowWater]:pedigree[B73] |  | 5.0999321 | 2.38009 | 2.14 |  | 0.0339 |
| pedigree[CML103]*(Mg-1171.6) |  | 0.0172354 | 0.008113 | 2.12 |  | 0.0354 |
| pedigree[IL14H]*(Mg-1171.6) |  | 0.0144585 | 0.007088 | 2.04 |  | 0.0432 |
| treatment[lowN]:pedigree[Ki11] |  | -7.608166 | 3.805812 | -2.00 |  | 0.0475 |
| treatment[lowN]:pedigree[CML103] |  | 5.7413248 | 2.879423 | 1.99 |  | 0.0481 |
| treatment[lowN]:pedigree[Tx303] |  | -8.8187 | 4.465624 | -1.97 |  | 0.0502 |
| pedigree[CML333]*(Mg-1171.6) |  | 0.0372381 | 0.019224 | 1.94 |  | 0.0547 |
| treatment[control]:pedigree[Hp301] |  | -6.638435 | 3.459138 | -1.92 |  | 0.0570 |
| treatment[lowN]:pedigree[Ky21] |  | -6.703884 | 3.55854 | -1.88 |  | 0.0616 |
| treatment[lowNlowWater]:pedigree[CML333] |  | 6.2504299 | 3.425267 | 1.82 |  | 0.0701 |
| treatment[lowN]:pedigree[CML277] |  | -8.17644 | 4.607831 | -1.77 |  | 0.0781 |
| treatment[lowWater]:pedigree[CML333] |  | 7.2503104 | 4.150172 | 1.75 |  | 0.0828 |
| treatment[lowWater]:pedigree[CML322] |  | 5.9025707 | 3.431238 | 1.72 |  | 0.0876 |
| pedigree[M37W]*(Mg-1171.6) |  | 0.0203955 | 0.011965 | 1.70 |  | 0.0905 |
| treatment[lowWater]:pedigree[Ki11] |  | -6.792208 | 4.050909 | -1.68 |  | 0.0958 |
| treatment[lowNlowWater]:pedigree[CML247] |  | 6.0774777 | 3.706076 | 1.64 |  | 0.1033 |
| treatment[lowNlowWater]:pedigree[CML277] |  | -5.64658 | 3.559347 | -1.59 |  | 0.1149 |
| treatment[lowN]:pedigree[hybrid] |  | -10.64586 | 6.718478 | -1.58 |  | 0.1153 |
| treatment[control]:pedigree[NC350] |  | 5.3729406 | 3.448183 | 1.56 |  | 0.1214 |
| treatment[lowN]:pedigree[CML69] |  | -6.186017 | 4.098545 | -1.51 |  | 0.1335 |
| pedigree[NC358]*(Mg-1171.6) |  | 0.0171605 | 0.011785 | 1.46 |  | 0.1476 |
| treatment[lowNlowWater]:pedigree[M162W] |  | -5.399667 | 3.73224 | -1.45 |  | 0.1502 |
| treatment[lowWater]:pedigree[IL14H] |  | -4.459353 | 3.088028 | -1.44 |  | 0.1509 |
| treatment[control]:pedigree[CML228] |  | 6.0168097 | 4.187728 | 1.44 |  | 0.1530 |
| treatment[lowWater]:pedigree[CML277] |  | -8.320529 | 6.012974 | -1.38 |  | 0.1686 |
| treatment[lowN]:pedigree[MS71] |  | -5.860235 | 4.2526 | -1.38 |  | 0.1704 |
| treatment[lowN]:pedigree[Mo18W] |  | -5.664518 | 4.28751 | -1.32 |  | 0.1886 |
| pedigree[Hp301]*(Mg-1171.6) |  | 0.0195449 | 0.01482 | 1.32 |  | 0.1894 |
| treatment[lowWater]:pedigree[M37W] |  | 4.7363041 | 3.609615 | 1.31 |  | 0.1916 |
| pedigree[CML277]*(Mg-1171.6) |  | -0.026122 | 0.019925 | -1.31 |  | 0.1920 |
| treatment[lowWater]:pedigree[B73] |  | 2.7263259 | 2.090483 | 1.30 |  | 0.1943 |
| pedigree[MS71]*(Mg-1171.6) |  | 0.0242238 | 0.019007 | 1.27 |  | 0.2046 |
| treatment[lowNlowWater]:pedigree[M37W] |  | -5.269258 | 4.172676 | -1.26 |  | 0.2087 |
| treatment[lowWater]:pedigree[CML69] |  | 3.5482804 | 2.840516 | 1.25 |  | 0.2137 |
| treatment[lowNlowWater]:pedigree[CML228] |  | 4.2685353 | 3.425805 | 1.25 |  | 0.2148 |
| treatment[control]:pedigree[IL14H] |  | -4.695123 | 3.849006 | -1.22 |  | 0.2246 |
| pedigree[M162W]*(Mg-1171.6) |  | 0.0287137 | 0.02371 | 1.21 |  | 0.2279 |
| treatment[control]:pedigree[Ky21] |  | -4.53168 | 3.769642 | -1.20 |  | 0.2313 |
| treatment[lowN]:pedigree[CML228] |  | -4.670857 | 3.888729 | -1.20 |  | 0.2317 |
| treatment[lowNlowWater]:pedigree[CML69] |  | 4.1453613 | 3.494857 | 1.19 |  | 0.2376 |
| pedigree[CML69]*(Mg-1171.6) |  | 0.010332 | 0.00877 | 1.18 |  | 0.2407 |
| pedigree[B97]*(Mg-1171.6) |  | 0.0163082 | 0.014271 | 1.14 |  | 0.2551 |
| treatment[lowN]:pedigree[CML247] |  | -4.156146 | 3.708877 | -1.12 |  | 0.2644 |
| treatment[lowNlowWater]:pedigree[Ki11] |  | -3.162483 | 2.841368 | -1.11 |  | 0.2676 |
| treatment[lowNlowWater]:pedigree[Ky21] |  | 4.5083778 | 4.068641 | 1.11 |  | 0.2697 |
| treatment[lowNlowWater]:pedigree[NC358] |  | -3.233853 | 2.921431 | -1.11 |  | 0.2702 |
| treatment[control]:pedigree[MS71] |  | 5.7053123 | 5.22335 | 1.09 |  | 0.2766 |
| treatment[lowNlowWater]:pedigree[MS71] |  | -3.083524 | 2.845531 | -1.08 |  | 0.2804 |
| treatment[lowN]:pedigree[Oh7B] |  | -3.882854 | 3.665982 | -1.06 |  | 0.2913 |
| treatment[lowNlowWater]:pedigree[Tx303] |  | -6.274756 | 6.660697 | -0.94 |  | 0.3478 |
| treatment[control]:pedigree[NC358] |  | -3.170485 | 3.421928 | -0.93 |  | 0.3558 |
| treatment[control]:pedigree[Tx303] |  | -3.945191 | 4.406002 | -0.90 |  | 0.3721 |
| treatment[lowNlowWater]:pedigree[IL14H] |  | -2.867545 | 3.2028 | -0.90 |  | 0.3721 |
| treatment[lowWater]:pedigree[M162W] |  | -4.283949 | 4.832419 | -0.89 |  | 0.3769 |
| treatment[control]:pedigree[hybrid] |  | -3.882664 | 4.389172 | -0.88 |  | 0.3779 |
| treatment[lowNlowWater]:pedigree[Oh43] |  | -6.036593 | 6.948158 | -0.87 |  | 0.3864 |
| treatment[lowNlowWater]:pedigree[NC350] |  | 3.4066613 | 4.122218 | 0.83 |  | 0.4100 |
| treatment[lowWater]:pedigree[Oh43] |  | -4.942113 | 5.992668 | -0.82 |  | 0.4109 |
| pedigree[Ky21]*(Mg-1171.6) |  | -0.010431 | 0.013656 | -0.76 |  | 0.4462 |
| treatment[lowNlowWater]:pedigree[Hp301] |  | -2.477311 | 3.468155 | -0.71 |  | 0.4762 |
| treatment[lowWater]:pedigree[Tx303] |  | -4.805345 | 6.863259 | -0.70 |  | 0.4850 |
| treatment[lowNlowWater]:pedigree[B97] |  | 3.4308812 | 5.073357 | 0.68 |  | 0.5000 |
| treatment[lowNlowWater]:pedigree[Mo18W] |  | -4.925892 | 7.737443 | -0.64 |  | 0.5254 |
| treatment[lowN]:pedigree[M37W] |  | -2.255297 | 3.625242 | -0.62 |  | 0.5349 |
| treatment[lowNlowWater]:pedigree[hybrid] |  | -4.93365 | 8.09998 | -0.61 |  | 0.5434 |
| pedigree[Oh43]*(Mg-1171.6) |  | 0.0126558 | 0.020998 | 0.60 |  | 0.5477 |
| treatment[control]:pedigree[Oh43] |  | -4.805821 | 7.995553 | -0.60 |  | 0.5488 |
| pedigree[hybrid]*(Mg-1171.6) |  | -0.010897 | 0.022507 | -0.48 |  | 0.6290 |
| treatment[control]:pedigree[CML322] |  | 5.0206881 | 10.40817 | 0.48 |  | 0.6303 |
| treatment[lowWater]:pedigree[MS71] |  | 1.4791407 | 3.086489 | 0.48 |  | 0.6325 |
| treatment[lowWater]:pedigree[NC358] |  | -2.173807 | 4.635545 | -0.47 |  | 0.6398 |
| treatment[lowN]:pedigree[B97] |  | 2.2824598 | 5.299184 | 0.43 |  | 0.6673 |
| treatment[control]:pedigree[CML247] |  | 1.2180717 | 2.848729 | 0.43 |  | 0.6696 |
| pedigree[NC350]*(Mg-1171.6) |  | 0.0119328 | 0.028474 | 0.42 |  | 0.6758 |
| treatment[lowNlowWater]:pedigree[CML322] |  | 2.9687898 | 7.207637 | 0.41 |  | 0.6810 |
| treatment[lowN]:pedigree[IL14H] |  | -1.135897 | 2.911947 | -0.39 |  | 0.6971 |
| treatment[control]:pedigree[M162W] |  | 1.9502739 | 5.073283 | 0.38 |  | 0.7012 |
| pedigree[CML322]*(Mg-1171.6) |  | 0.0144828 | 0.044668 | 0.32 |  | 0.7462 |
| treatment[lowWater]:pedigree[Ky21] |  | 1.3680424 | 4.224431 | 0.32 |  | 0.7465 |
| treatment[control]:pedigree[M37W] |  | 1.2140313 | 3.927341 | 0.31 |  | 0.7577 |
| treatment[lowN]:pedigree[Oh43] |  | 1.8941858 | 6.558007 | 0.29 |  | 0.7731 |
| pedigree[CML247]*(Mg-1171.6) |  | 0.0050048 | 0.017529 | 0.29 |  | 0.7757 |
| treatment[lowN]:pedigree[CML322] |  | 1.7814551 | 7.384016 | 0.24 |  | 0.8097 |
| treatment[lowWater]:pedigree[CML247] |  | -0.828087 | 3.530321 | -0.23 |  | 0.8149 |
| treatment[lowWater]:pedigree[Oh7B] |  | 0.9850583 | 4.220952 | 0.23 |  | 0.8158 |
| treatment[control]:pedigree[CML69] |  | -0.799513 | 3.87653 | -0.21 |  | 0.8369 |
| treatment[lowWater]:pedigree[hybrid] |  | 0.9996511 | 4.992493 | 0.20 |  | 0.8416 |
| treatment[lowN]:pedigree[M162W] |  | 0.9764101 | 5.34782 | 0.18 |  | 0.8554 |
| treatment[lowWater]:pedigree[Hp301] |  | -0.436508 | 2.490625 | -0.18 |  | 0.8611 |
| treatment[control]:pedigree[Mo18W] |  | -1.201617 | 6.886369 | -0.17 |  | 0.8617 |
| pedigree[CML228]*(Mg-1171.6) |  | 0.0041934 | 0.025475 | 0.16 |  | 0.8695 |
| treatment[control]:pedigree[Ki11] |  | -0.635076 | 4.318581 | -0.15 |  | 0.8833 |
| pedigree[Mo18W]*(Mg-1171.6) |  | 0.004778 | 0.034362 | 0.14 |  | 0.8896 |
| treatment[lowWater]:pedigree[CML228] |  | -0.720066 | 5.383841 | -0.13 |  | 0.8938 |
| pedigree[Oh7B]*(Mg-1171.6) |  | -0.001376 | 0.011002 | -0.13 |  | 0.9006 |
| treatment[lowNlowWater]:pedigree[Oh7B] |  | -0.367354 | 3.968884 | -0.09 |  | 0.9264 |
| treatment[lowWater]:pedigree[NC350] |  | 0.6761526 | 7.920459 | 0.09 |  | 0.9321 |
| treatment[lowN]:pedigree[Hp301] |  | -0.259203 | 3.939047 | -0.07 |  | 0.9476 |
| treatment[lowN]:pedigree[CML333] |  | 0.4493017 | 7.508162 | 0.06 |  | 0.9524 |
| treatment[lowWater]:pedigree[B97] |  | -0.241506 | 4.373219 | -0.06 |  | 0.9560 |
| treatment[control]:pedigree[B97] |  | -0.17196 | 3.236819 | -0.05 |  | 0.9577 |
| pedigree[Ki11]*(Mg-1171.6) |  | -0.000545 | 0.013451 | -0.04 |  | 0.9677 |
| treatment[lowN]:pedigree[NC350] |  | 0.0848968 | 9.484263 | 0.01 |  | 0.9929 |
| pedigree[Tx303]*(Mg-1171.6) |  | 0.0000391 | 0.034776 | 0.00 |  | 0.9991 |

**pedigree*Mg**

**pedigree[treatment]**

**LSMeans Differences Tukey HSD**

α=

0.050	Q=

4.42184

| **Level** |  |  |  | **Least Sq Mean** |
| --- | --- | --- | --- | --- |
| [lowN]P39 | A | B | C | 77.050317 |
| [lowWater]CML52 | A | B | C | 40.442539 |
| [lowNlowWater]CML52 | A | B | C | 33.463357 |
| [control]CML52 | A | B | C | 32.707308 |
| [control]CML103 | A | B | C | 32.311480 |
| [lowN]B73 | A |  |  | 32.170306 |
| [lowN]CML52 | A | B | C | 31.715091 |
| [control]B73 | A | B |  | 30.910533 |
| [lowNlowWater]CML103 | A | B | C | 29.534292 |
| [control]CML333 | A | B | C | 28.526337 |
| [lowWater]CML103 | A | B | C | 28.033145 |
| [lowWater]CML333 | A | B | C | 27.682025 |
| [lowNlowWater]CML333 | A | B | C | 26.682145 |
| [lowNlowWater]CML247 | A | B | C | 26.509192 |
| [control]CML228 | A | B | C | 26.448524 |
| [lowWater]CML322 | A | B | C | 26.334285 |
| [lowN]CML103 | A | B | C | 26.173039 |
| [control]MS71 | A | B | C | 26.137027 |
| [control]NC350 | A | B | C | 25.804655 |
| [lowNlowWater]B73 | A | B | C | 25.531647 |
| [control]CML322 | A | B | C | 25.452403 |
| [lowWater]M37W | A | B | C | 25.168019 |
| [lowNlowWater]Ky21 | A | B | C | 24.940092 |
| [lowNlowWater]CML228 | A | B | C | 24.700250 |
| [lowNlowWater]CML69 | A | B | C | 24.577076 |
| [lowWater]CML69 | A | B | C | 23.979995 |
| [lowNlowWater]B97 | A | B | C | 23.862596 |
| [lowNlowWater]NC350 | A | B | C | 23.838376 |
| [lowNlowWater]CML322 | A | B | C | 23.400504 |
| [lowWater]B73 | A | B | C | 23.158041 |
| [lowN]B97 | A | B | C | 22.714174 |
| [control]M162W | A | B | C | 22.381988 |
| [lowN]Oh43 | A | B | C | 22.325900 |
| [lowN]CML322 | A | B | C | 22.213170 |
| [lowWater]MS71 | A | B | C | 21.910855 |
| [lowWater]Ky21 | A | B | C | 21.799757 |
| [control]CML247 | A | B | C | 21.649786 |
| [control]M37W | A | B | C | 21.645746 |
| [lowWater]hybrid | A | B | C | 21.431366 |
| [lowWater]Oh7B | A | B | C | 21.416773 |
| [lowN]M162W | A | B | C | 21.408125 |
| [lowWater]NC350 | A | B | C | 21.107867 |
| [lowN]CML333 | A | B | C | 20.881016 |
| [lowN]NC350 | A | B | C | 20.516611 |
| [control]B97 | A | B | C | 20.259755 |
| [lowWater]B97 | A | B | C | 20.190209 |
| [lowN]Hp301 | A | B | C | 20.172512 |
| [lowNlowWater]Oh7B | A | B | C | 20.064361 |
| [lowWater]Hp301 | A | B | C | 19.995207 |
| [control]Ki11 | A | B | C | 19.796638 |
| [lowWater]CML228 | A | B | C | 19.711648 |
| [control]CML69 | A | B | C | 19.632202 |
| [lowWater]CML247 | A | B | C | 19.603628 |
| [lowN]IL14H | A | B | C | 19.295817 |
| [control]Mo18W | A | B | C | 19.230098 |
| [lowWater]NC358 | A | B | C | 18.257908 |
| [lowN]M37W | A | B | C | 18.176417 |
| [lowNlowWater]Hp301 | A | B | C | 17.954403 |
| [lowNlowWater]IL14H | A | B | C | 17.564169 |
| [lowNlowWater]MS71 | A | B | C | 17.348191 |
| [lowNlowWater]Ki11 | A | B | C | 17.269232 |
| [control]NC358 | A | B | C | 17.261229 |
| [lowNlowWater]NC358 | A | B | C | 17.197862 |
| [control]hybrid | A | B | C | 16.549051 |
| [lowN]Oh7B | A | B | C | 16.548861 |
| [control]Tx303 | A | B | C | 16.486523 |
| [lowN]CML247 | A | B | C | 16.275568 |
| [lowWater]M162W | A | B | C | 16.147766 |
| [lowWater]IL14H | A | B | C | 15.972362 |
| [control]Ky21 | A | B | C | 15.900035 |
| [lowN]CML228 | A | B | C | 15.760857 |
| [control]IL14H | A | B | C | 15.736592 |
| [lowWater]Tx303 | A | B | C | 15.626370 |
| [control]Oh43 | A | B | C | 15.625894 |
| [lowNlowWater]Mo18W | A | B | C | 15.505822 |
| [lowNlowWater]hybrid | A | B | C | 15.498065 |
| [lowWater]Oh43 | A | B | C | 15.489602 |
| [lowNlowWater]M37W | A | B | C | 15.162456 |
| [lowNlowWater]M162W | A | B | C | 15.032048 |
| [lowNlowWater]CML277 | A | B | C | 14.785135 |
| [lowN]Mo18W | A | B | C | 14.767196 |
| [lowN]MS71 | A | B | C | 14.571479 |
| [lowNlowWater]Oh43 | A | B | C | 14.395122 |
| [lowN]CML69 | A | B | C | 14.245698 |
| [lowNlowWater]Tx303 | A | B | C | 14.156959 |
| [control]Hp301 |  | B | C | 13.793279 |
| [lowN]Ky21 |  | B | C | 13.727830 |
| [lowWater]Ki11 | A | B | C | 13.639507 |
| [lowN]NC358 |  |  | C | 13.455462 |
| [lowWater]Tzi8 |  | B | C | 13.164225 |
| [control]Oh7B |  |  | C | 13.015845 |
| [lowN]Ki11 |  | B | C | 12.823549 |
| [lowN]CML277 | A | B | C | 12.255275 |
| [lowWater]CML277 | A | B | C | 12.111185 |
| [lowNlowWater]Tzi8 |  |  | C | 11.819514 |
| [lowN]Tx303 | A | B | C | 11.613014 |
| [lowN]Tzi8 |  |  | C | 10.581436 |
| [lowN]hybrid | A | B | C | 9.785856 |
| [control]Tzi8 |  | B | C | 9.770422 |
| [lowWater]Mo18W |  |  | C | 8.418582 |
| [control]CML277 | A | B | C | 3.759516 |

Levels not connected by same letter are significantly different.
