# Supplementary material for: Abiotic stress growth conditions induce different responses in kernel iron concentration across genotypically distinct maize inbred varieties
Source: Front Plant Sci. 2013 Dec 4;4:488. doi: 10.3389/fpls.2013.00488 (PMC3850239; doi:10.3389/fpls.2013.00488)
Supplement: Data Sheet 1 — Grain mineral and physiological data for each genotype- treatment combination is grouped into separate sheets in the xls file. Inbred genotypes are listed by their letter-number codes using the maize genetics naming conventions. Mineral concentration data from ICP analysis includes experimental plot-specific entry numbers, the inbred ID, the ear number (as some measurements required kernels from more than one ear), while kernel and cob data are presented as entry means. [file DataSheet1.ZIP › 68761_Stapleton_Data_Sheet_6.DOC]

Supplemental File 6 Correlation between Kernel Iron and Magnesium Concentration

***Experiment-wide and treatment-specific pairwise correlations between iron and magnesium concentration.***

|  | | **variable** | **by variable** | **correlation** | **count** | **P value** |
| --- | --- | --- | --- | --- | --- | --- |
| Overall correlation between iron and magnesium concentration | | Mg | Fe | 0.3162 | 263 | P<0.0001** |
|  | | | | | | |
| Correlation in stress treatment environments | Control | Mg | Fe | 0.3423 | 64 | 0.0056** |
|  | Drought | Mg | Fe | 0.2000 | 65 | 0.1102 |
|  | Low nitrogen | Mg | Fe | 0.4267 | 66 | 0.0004** |
|  | Combined | Mg | Fe | 0.3146 | 68 | 0.009** |
